# Fecundability in Association With Everyday and Lifetime Discrimination

**DOI:** 10.1001/jamanetworkopen.2025.20597

**Published:** 2025-07-14

**Authors:** Ugochinyere Vivian Ukah, Sharonda M. Lovett, Renée Boynton-Jarrett, Jasmine Abrams, Amelia K. Wesselink, Molly N. Hoffman, David R. Williams, Lauren A. Wise, Collette N. Ncube

**Affiliations:** 1 Department of Medicine, McGill University, Montreal, Quebec, Canada; 2 Department of Epidemiology, Boston University School of Public Health, Boston, Massachusetts; 3 Division of Health Services Research, Department of Pediatrics, Boston Medical Center, Boston, Massachusetts; 4 Department of Social and Behavioral Sciences, Yale University School of Public Health, New Haven, Connecticut; 5 Department of Social and Behavioral Sciences, Harvard T.H. Chan School of Public Health, Boston, Massachusetts

## Abstract

**Importance:**

There are well-documented racial and socioeconomic disparities in fertility outcomes, yet little is known about the association between discrimination and fecundability.

**Objective:**

To examine the association of self-reported experiences of everyday and lifetime discrimination with fecundability.

**Design, Setting, and Participants:**

This cohort study included data from the Pregnancy Study Online, a web-based prospective preconception cohort study that examines whether lifestyle factors are associated with fertility and pregnancy. Female participants aged 21 to 45 years in the US and Canada who were attempting pregnancy were recruited online from June 2013 to January 2023 and followed up for up to 12 months.

**Exposures:**

Everyday discrimination (eg, disrespect, harassment) and lifetime discrimination (eg, on the job, by police) as measured using adapted versions of Williams’ Everyday Discrimination Scale and Major Experiences of Discrimination scale. Summary score variables were created for frequency of everyday discrimination (ranging from 0 to ≥7, with scores of ≥7 indicating very high everyday discrimination) and for the number of lifetime discrimination types (ranging from 0 to ≥3, with ≥3 indicating greater lifetime discrimination). Attributions of and responses to discrimination were also examined.

**Main Outcomes and Measures:**

Fecundability, the per-cycle probability of conception, was measured using bimonthly follow-up questionnaires. Proportional probabilities regression models were used to estimate fecundability ratios (FRs) and 95% CIs across categories of everyday and lifetime discrimination. Adjustments were made for potential confounders and stratified analyses by race and ethnicity.

**Results:**

Among 6578 female participants (mean [SD] age, 30.5 [3.9] years), 1164 (17.7%) reported everyday discrimination scores of 7 or more, and 3144 (47.8%) reported 1 or more experiences of lifetime discrimination. In terms of race and ethnicity, 375 participants (5.7%) identified as Hispanic, 110 (1.7%) as non-Hispanic Black, 5701 (86.7%) as non-Hispanic White, and 392 (6.0%) as non-Hispanic other race (including American Indian or Alaska Native, Asian, Native Hawaiian or Other Pacific Islander, and multiracial or other race). Everyday discrimination was associated with reduced fecundability (score ≥7 vs 0: FR, 0.82 [95% CI, 0.75-0.90]; especially harassment [few times or more per month] vs none: FR, 0.74 [95% CI, 0.64-0.87]). Any experience (vs none) of lifetime discrimination was associated with decreased fecundability (FR, 0.93 [95% CI, 0.88-0.99]), especially discrimination when receiving medical care (FR, 0.88 [95% CI, 0.81-0.95]) and when on the job (FR, 0.94 [95% CI, 0.88-0.99]) vs no such discrimination. Reduced fecundability was mostly observed among White participants who experienced everyday discrimination (everyday discrimination score ≥7 vs 0: FR, 0.79 [95% CI, 0.71-0.87]) and among racial and ethnic minority individuals who experienced any lifetime discrimination (FR, 0.80 (95% CI, 0.68-0.94]) compared with no such discrimination. Participants who attributed their discrimination to race or ethnicity (lifetime discrimination score of 2 vs 0: FR, 0.66 [95% CI, 0.52-0.83]) or who kept quiet and accepted their experiences (everyday discrimination score ≥7 vs 0: FR, 0.76 [95% CI, 0.63-0.92]) were generally more likely to have reduced fecundability.

**Conclusions and Relevance:**

The findings of this cohort study suggest that everyday and lifetime experiences of discrimination were associated with reduced fecundability. Associations varied by frequency and type of discrimination, highlighting the need for more research on the possible reproductive health effects of unfair treatment.

## Key Points

**Question:**

To what extent are self-reported experiences of discrimination associated with reduced fecundability among female adults residing in the US and Canada?

**Findings:**

In this cohort study of 6578 female participants, experiences of everyday and lifetime discrimination were associated with reduced fecundability. Associations between everyday discrimination and fecundability were mostly observed among White participants, while associations between lifetime discrimination and fecundability were mostly observed among racial and ethnic minority participants.

**Meaning:**

These findings suggest that discrimination may be a determinant of reduced fecundability, highlighting the need for more research on the effects of discrimination on reproductive health.

## Teaser

This cohort study of female adults assesses whether self-reported experiences of everyday and lifetime discrimination are associated with reduced fecundability in the US and Canada.

## Introduction

Infertility affects millions of couples in the US and Canada^1^ and has adverse psychosocial and financial outcomes.^1,2^ Some US studies indicate that racially and ethnically minoritized populations have higher rates of infertility and are less likely to achieve their fertility goals than non-Hispanic White individuals,^3,4,5,6,7^ but there is sparse literature on disparities in infertility in Canada.^8,9,10^ Discrimination is linked with adverse perinatal outcomes^11,12,13,14,15,16^ including preterm birth,^17,18^ low birthweight,^17,18^ and small-for-gestational age.^17,19,20,21,22^ However, no studies, to our knowledge, have examined the association between discrimination and fertility.

Interpersonal racism and other forms of discrimination can reduce access to reproductive health care, adversely affect quality of care, and promote distrust in the medical system.^17,23,24^ Furthermore, discrimination may increase stress and weathering, which may affect fecundability.^5,11,17,24,25,26^ Fecundability, a sensitive marker of fertility, is defined as the probability of conception within 1 menstrual cycle given unprotected intercourse.^27,28,29^ Given existing knowledge gaps, we investigated the association of everyday and lifetime discrimination with fecundability in this preconception cohort study.

## Methods

### Study Design and Population

We analyzed data from participants enrolled between June 2013 and January 2023 in the Pregnancy Study Online, a preconception cohort study.^30^ Eligible participants self-identified as female and were aged 21 to 45 years, were US or Canadian residents, were not using contraception or fertility treatments at enrollment, and were actively trying to conceive with a male partner. Participants completed a baseline questionnaire on sociodemographic, behavioral, and medical characteristics and shorter follow-up questionnaires every 8 weeks for up to 12 months or until conception, whichever occurred first.

The Boston University Institutional Review Board approved the study protocol. All participants provided informed consent online. The study followed the Strengthening the Reporting of Observational Studies in Epidemiology (STROBE) reporting guideline.

### Assessment of Discrimination

The primary exposures were everyday discrimination and lifetime discrimination measured using adapted versions of Williams’ Everyday Discrimination Scale and Major Experiences of Discrimination scales.^11,31^ These instruments were included on Pregnancy Study Online’s Life Course Experiences Questionnaire (LCEQ), a supplemental questionnaire launched in July 2019 and completed 30 days after enrollment.^32^ Participants who were recruited before July 2019 and had active email addresses were recontacted and asked to complete the LCEQ retrospectively.

For everyday discrimination, participants reported the type (eg, treated with less courtesy or respect than other people) and frequency (eg, a few times a month) of specific incidents in their day-to-day life (eTable 1 in Supplement 1).^11^ For lifetime discrimination, participants reported if they had ever been treated unfairly on the job, in housing, by police, in the courts, at school, or while getting medical care.

Participants reported what they perceived as the main reason for their experiences of discrimination (choosing all that applied): race or ethnicity, sex or gender, educational or income level, and other (eTable 2 in Supplement 1). Finally, based on questions developed by Krieger et al,^33^ we asked participants who reported discrimination how they responded when treated unfairly (choosing 1): keep it to yourself (quiet) or talk to other people about it (talk) and (choosing 1): accept it as a fact of life (accept) or try to do something about it (act).

### Assessment of Fecundability

On the baseline questionnaire, participants reported the date of their last menstrual period (LMP), regularity, usual length of menstrual cycle, and number of cycles trying to conceive. Among those with irregular cycles, we estimated cycle length based on baseline and prospectively reported LMP dates. On follow-up questionnaires, we collected information on pregnancy status and pregnancy loss since the last questionnaire. More than 96.0% of pregnancies were confirmed using home pregnancy tests. Among participants lost to follow-up, we sought pregnancy information using telephone interviews, online baby announcements and registries, and birth registries in 8 states (where about 50% of the US participants resided).^34^ We calculated time to pregnancy (cycles)^35^ as follows: cycles of pregnancy attempt at study entry + ([LMP date from the most recent follow-up questionnaire − date of the baseline questionnaire completion]/cycle length) + 1.

### Assessment of Covariates

For potential confounders, we considered determinants of fecundability^4,36,37,38,39^ that were reported on the baseline questionnaire (age, parental educational level, and race and ethnicity) and on the LCEQ (adverse childhood experiences [ACEs],^40,41^ childhood financial hardship). We grouped self-identified race and ethnicity (conceptualized as a socially constructed variable^42^) based on US Census categories^43^: Hispanic, non-Hispanic Black (hereinafter Black), non-Hispanic White (hereinafter White), and non-Hispanic other race (including American Indian or Alaska Native, Asian, Native Hawaiian or Other Pacific Islander, and multiracial or other race]). We defined racial and ethnic minority individuals as all participants who did not identify as White.

We derived a measure of ACEs^40,41^ by summing *yes* responses across 8 experiences occurring before age 18 years (ranging from 0 to 8, with higher scores indicating more ACEs): household mental illness, household substance use, household incarceration, parental separation or divorce, parental intimate-partner violence, physical abuse, emotional abuse, and sexual abuse. We defined childhood financial hardship as not having enough money to pay for food, rent, or mortgage; having to borrow money to pay for medical expenses; or receiving public assistance or welfare before age 18 years (yes vs no). The latter measure was partially derived from the sociocultural module of the US Department of Health and Human Services’ Breast Cancer Core Questionnaire.^44,45,46,47,48^

### Exclusions

Among 16 972 enrolled participants, we excluded participants whose baseline LMP was more than 6 months before enrollment to reduce bias due to menstrual irregularity or unconfirmed pregnancy (n = 166), or those who had missing or implausible LMP data (n = 92). We also excluded persons who had been attempting pregnancy for more than 6 cycles at enrollment to reduce selection bias and reverse causation bias (n = 3320). Including participants with fewer than 6 cycles at enrollment helped to ensure that they were more likely to have just started trying to conceive and had not yet experienced fertility problems at study entry, thereby reducing the potential for both the exposure and outcome to possibly affect participation in the study (ie, selection bias). This also allowed us to capture participants earlier in their fertility journey and follow them over the full spectrum. Finally, we excluded 6816 nonrespondents to the LCEQ. In general, LCEQ respondents were more likely than nonrespondents to be White, college-educated, and have a higher income level (eTable 3 in Supplement 1).

### Statistical Analysis

We used proportional probabilities regression models^49^ to estimate fecundability ratios (FRs) and 95% CIs for the associations of everyday discrimination and lifetime discrimination with fecundability. We used the Andersen-Gill data structure with 1 observation per cycle to account for left truncation.^50,51,52^ Participants contributed at-risk menstrual cycles to the analysis from enrollment until a reported pregnancy or censoring event (fertility treatment initiation, cessation of pregnancy attempts, loss to follow-up [date of last completed questionnaire], 12 cycles of attempt time, or still participating), whichever came first.

We created binary indicators (a few times or more per month vs none for specific type) and a summary score variable for everyday discrimination by assigning a score to the Likert scale (0 = never, 1 = less than once a year, 2 = a few times a year, 3 = a few times a month, 4 = at least once a week, and 5 = almost every day), summing across each type of discrimination (disrespect, poor service, not smart, afraid of you, and harassed) and ranging from 0 to 25, with higher scores indicating more everyday discrimination. We then divided this score variable into 5 categories based on the cohort distribution: none (0 [reference category]), low (1-2), medium (3-4), high (5-6), and very high (≥7). We also generated restricted cubic splines of associations between everyday discrimination (summary variable) and fecundability.^53^ We created binary indicators (yes vs no) and a summary score variable for the number of lifetime discrimination experiences (job, housing, police, courts, school, and medical care), with scores ranging from 0 to 3 or more, in which 3 or more indicates greater lifetime discrimination. In all analyses, we stratified by race and ethnicity (White compared with racial and ethnic minority individuals).

We used a directed acyclic graph (eFigure in Supplement 1)^54^ to guide selection of potential confounders. The first model (minimally adjusted) included age (<25, 25-29, 30-34, and ≥35 years) and race and ethnicity (Black, Hispanic, White, and multiracial or other race), while the second model (fully adjusted) further adjusted for parental educational level (≤12, 13-15, 16, and ≥17 years), ACEs (yes vs no), and childhood financial hardship (yes vs no).

We cross-classified the summary variables for everyday and lifetime discrimination with participants’ primary attribution for discrimination (race and ethnicity vs other attribution). We then examined the association between the cross-classified variable and fecundability. We used a similar approach for response type collapsed into quiet and accept and talk and act. We repeated these analyses excluding White participants who reported race or ethnicity as an attribution to avoid attribution of discrimination to reverse racism.

In sensitivity analyses, we performed cross-classified analyses for attributions of sex or gender or any attribution other than sex or gender. We also stratified analyses by retrospective vs prospective LCEQ completion, pregnancy-attempt time at enrollment (<3 vs 3-6 cycles), country of residence at enrollment (US vs Canada) to examine potential transnational differences, and LCEQ completion before vs after June 2020 to examine potential differences associated with major world events (eg, the COVID-19 pandemic, death of George Floyd). For all analyses, we interpreted our findings by considering the size and direction of the point estimates, CI widths, and consistency of results across models rather than relying solely on binary statistical significance testing.

Missingness ranged from 0.3% (intercourse frequency) to 21.6% (health insurance [added to baseline questionnaire in January 2018]). We used fully conditional specification methods^55^ to multiply impute missing data on exposures, covariates, and pregnancy status by creating 20 imputed datasets. Analyses were performed using SAS, version 9.4 (SAS Institute Inc).

## Results

The analytic sample comprised 6578 female participants (mean [SD] age, mean [SD] age, 30.5 [3.9] years), of whom 3299 (50.2%) completed the LCEQ retrospectively, and 3279 (49.8%) completed the LCEQ prospectively. Of the participants who contributed at-risk menstrual cycles from enrollment until a pregnancy or censoring event, 559 (8.5%) reported fertility treatment initiation, 173 (2.6%) reported cessation of pregnancy attempts, 493 (7.5%) were lost to follow-up, 784 (11.9%) reported 12 cycles of attempt time, and 96 (1.5%) still participated.

A total of 110 participants (1.7%) self-identified as Black, 375 (5.7%) as Hispanic, 5701 (86.7%) as White, and 392 (6.0%) as multiracial or other race. Participants who completed the LCEQ retrospectively vs prospectively were similar in terms of age, race and ethnicity, and educational level (eTable 4 in Supplement 1). Participants lost to follow-up were similar to those included with respect to age and parity but were more likely to identify as racial and ethnic minority individuals and have a lower educational or income level (eTable 4 in Supplement 1).

The frequency of any everyday discrimination was 5421 (82.4%), of whom 1164 (17.7%) reported scores of 7 or more, and 3144 (47.8%) reported any lifetime discrimination. Participants who identified as racial and ethnic minority individuals, had fewer than 16 years of education, had an annual income less than $50 000, or who currently smoked reported higher levels of everyday and lifetime discrimination (Table 1).

**Table 1.** Baseline Characteristics of Participants by Discrimination^a^

Characteristic	Participant discrimination type (N = 6578)								
	Everyday^b^					Lifetime^c^			
	0	1-2	3-4	5-6	≥7	0	1	2	≥3
Total	1157 (17.6)	1353 (20.6)	1635 (24.9)	1269 (19.3)	1164 (17.7)	3434 (52.2)	1569 (23.9)	886 (13.5)	689 (10.5)
Age, mean (SD), y	30.4 (3.8)	30.6 (3.9)	30.6 (3.8)	30.8 (4.0)	30.2 (4.1)	30.4 (3.7)	30.8 (3.9)	30.5 (4.1)	30.8 (4.3)
Married	1069 (92.4)	1241 (91.7)	1490 (91.1)	1115 (87.9)	995 (85.5)	3163 (92.1)	1411 (89.9)	779 (87.9)	557 (80.8)
Race and ethnicity									
Hispanic	55 (4.8)	71 (5.2)	79 (4.8)	78 (6.1)	92 (7.9)	170 (5.0)	89 (5.7)	52 (5.9)	64 (9.3)
Non-Hispanic Black	6 (0.5)	8 (0.6)	24 (1.5)	21 (1.7)	51 (4.4)	23 (0.7)	22 (1.4)	34 (3.8)	31 (4.5)
Non-Hispanic White	1044 (90.2)	1209 (89.4)	1435 (87.8)	1081 (85.2)	932 (80.1)	3082 (89.7)	1366 (87.1)	723 (81.6)	530 (76.9)
Non-Hispanic other^d^	52 (4.5)	65 (4.8)	97 (5.9)	89 (7.0)	89 (7.6)	159 (4.6)	92 (5.9)	77 (8.7)	64 (9.3)
Geographic region of residence in adulthood									
Northeastern US	283 (24.5)	304 (22.5)	356 (21.8)	280 (22.1)	232 (19.9)	803 (23.4)	343 (21.9)	192 (21.7)	117 (17.0)
Southern US	241 (20.8)	292 (21.6)	382 (23.4)	277 (21.8)	259 (22.3)	716 (20.9)	346 (22.1)	208 (23.5)	181 (26.3)
Midwestern US	270 (23.3)	296 (21.9)	336 (20.6)	280 (22.1)	279 (24.0)	830 (24.2)	308 (19.6)	172 (19.4)	151 (21.9)
Western US	192 (16.6)	263 (19.4)	279 (17.1)	223 (17.6)	209 (18.0)	576 (16.8)	314 (20.0)	149 (16.8)	127 (18.4)
Canada	171 (14.8)	198 (14.6)	282 (17.2)	209 (16.5)	185 (15.9)	509 (14.8)	258 (16.4)	165 (18.6)	113 (16.4)
Educational attainment, y									
≤12	29 (2.5)	20 (1.5)	36 (2.2)	49 (3.9)	50 (4.3)	73 (2.1)	35 (2.2)	36 (4.1)	40 (5.8)
13-15	144 (12.4)	146 (10.8)	182 (11.1)	167 (13.2)	264 (22.7)	358 (10.4)	187 (11.9)	178 (20.1)	180 (26.1)
16	426 (36.8)	430 (31.8)	533 (32.6)	450 (35.5)	364 (31.3)	1161 (33.8)	548 (34.9)	285 (32.2)	209 (30.3)
≥17	558 (48.2)	757 (55.9)	884 (54.1)	603 (47.5)	486 (41.8)	1842 (53.6)	799 (50.9)	387 (43.7)	260 (37.7)
Annual household income, $									
<50 000	107 (9.2)	134 (9.9)	173 (10.6)	169 (13.3)	211 (18.1)	308 (9.0)	175 (11.2)	138 (15.6)	173 (25.1)
50 000-99 999	405 (35.0)	401 (29.6)	566 (34.6)	426 (33.6)	425 (36.5)	1080 (31.5)	558 (35.6)	340 (38.4)	245 (35.6)
100 000-149 999	374 (32.3)	434 (32.1)	484 (29.6)	364 (28.7)	301 (25.9)	1089 (31.7)	458 (29.2)	241 (27.2)	169 (24.5)
≥150 000	271 (23.4)	384 (28.4)	412 (25.2)	310 (24.4)	227 (19.5)	957 (27.9)	378 (24.1)	167 (18.8)	102 (14.8)
Body mass index, mean (SD)^e^	26.8 (6.6)	26.0 (5.9)	26.7 (6.6)	27.3 (7.0)	28.7 (7.7)	26.1 (6.1)	27.2 (6.7)	28.6 (7.6)	29.5 (7.9)
Current alcohol intake ≥14 drinks/wk	23 (2.0)	33 (2.4)	38 (2.3)	32 (2.5)	29 (2.5)	75 (2.2)	41 (2.6)	19 (2.1)	20 (2.9)
Current smoker	45 (3.9)	42 (3.1)	69 (4.2)	61 (4.8)	92 (7.9)	121 (3.5)	81 (5.2)	38 (4.3)	69 (10.0)
Age at menarche <12 y	250 (21.6)	291 (21.5)	372 (22.8)	324 (25.5)	333 (28.6)	754 (22.0)	388 (24.7)	217 (24.5)	211 (30.6)
Parous	416 (36.0)	425 (31.4)	499 (30.5)	389 (30.7)	383 (32.9)	1040 (30.3)	496 (31.6)	298 (33.6)	278 (40.3)
No. of previous births									
0	741 (64.0)	928 (68.6)	1136 (69.5)	880 (69.3)	781 (67.1)	2394 (69.7)	1073 (68.4)	588 (66.4)	411 (59.7)
1	280 (24.2)	304 (22.5)	383 (23.4)	271 (21.4)	266 (22.9)	779 (22.7)	353 (22.5)	210 (23.7)	162 (23.5)
≥2	136 (11.8)	121 (8.9)	116 (7.1)	118 (9.3)	117 (10.1)	261 (7.6)	143 (9.1)	88 (9.9)	116 (16.8)
Private health insurance	988 (85.4)	1196 (88.4)	1395 (85.3)	1064 (83.8)	925 (79.5)	3001 (87.4)	1341 (85.5)	707 (79.8)	519 (75.3)
Prenatal vitamin, folate, or multivitamin use	962 (83.1)	1156 (85.4)	1397 (85.4)	1077 (84.9)	936 (80.4)	2964 (86.3)	1313 (83.7)	725 (81.8)	526 (76.3)
Intercourse frequency ≥2-3 times/wk	659 (57.0)	761 (56.2)	896 (54.8)	713 (56.2)	683 (58.7)	1945 (56.6)	875 (55.8)	493 (55.6)	399 (57.9)
Last method of hormonal contraception^f^	411 (35.5)	465 (34.4)	562 (34.4)	416 (32.8)	416 (35.7)	1221 (35.6)	533 (34.0)	308 (34.8)	208 (30.2)

^a^ Data are from the Pregnancy Study Online cohort that includes female participants, aged 21 to 45 years, in the US and Canada who were recruited online from June 2013 to January 2023. Data are presented as the No. (%) of participants unless otherwise indicated.

^b^ The summary variable for everyday discrimination (disrespect, poor service, not smart, afraid of you, or harassed) was created after assigning a score to each Likert scale and summing type of everyday discrimination (ranging from 0 to 25, in which 0 = never, 1 = less than once a year, 2 = a few times a year, 3 = a few times a month, 4 = at least once a week, and 5 = almost every day). The score variable was then divided into 5 categories based on the cohort distribution: none (0 [reference category]), low (1-2), medium (3-4), high (5-6), and very high (≥7).

^c^ Reported lifetime discrimination in any of the following settings: on the job, in housing, by police, in the courts, at school, or getting medical care. Numbers of lifetime discrimination events range from 0 to 3 or more, with 3 or more indicating greater lifetime discrimination.

^d^ Includes American Indian or Alaska Native, Asian, Native Hawaiian or Other Pacific Islander, and multiracial or other race.

^e^ Calculated as weight in kilograms divided by height in meters squared.

^f^ Includes oral contraceptive pill, contraceptive patch, injectable, vaginal ring, implantable rods, or hormone-containing intrauterine device.

Across all racial and ethnic groups, “people acting as if they thought you were not smart” was the most reported form of everyday discrimination (4254 [64.7%]), and job discrimination was the most frequent lifetime experience (2274 [34.6%]) (eTable 1 in Supplement 1). Among those who reported discrimination, race or ethnicity was the most common attribution among Black participants (95 [88.8%]), whereas sex or gender was the most common attribution among other racial and ethnic groups (ranging from 74.2% to 80.2%) (eTable 2 in Supplement 1). The most frequent response types were quiet and accept (2068 [37.0%]) and talk and accept (1902 [34.0%]).

### Everyday Discrimination and Fecundability

**Figure 1.**
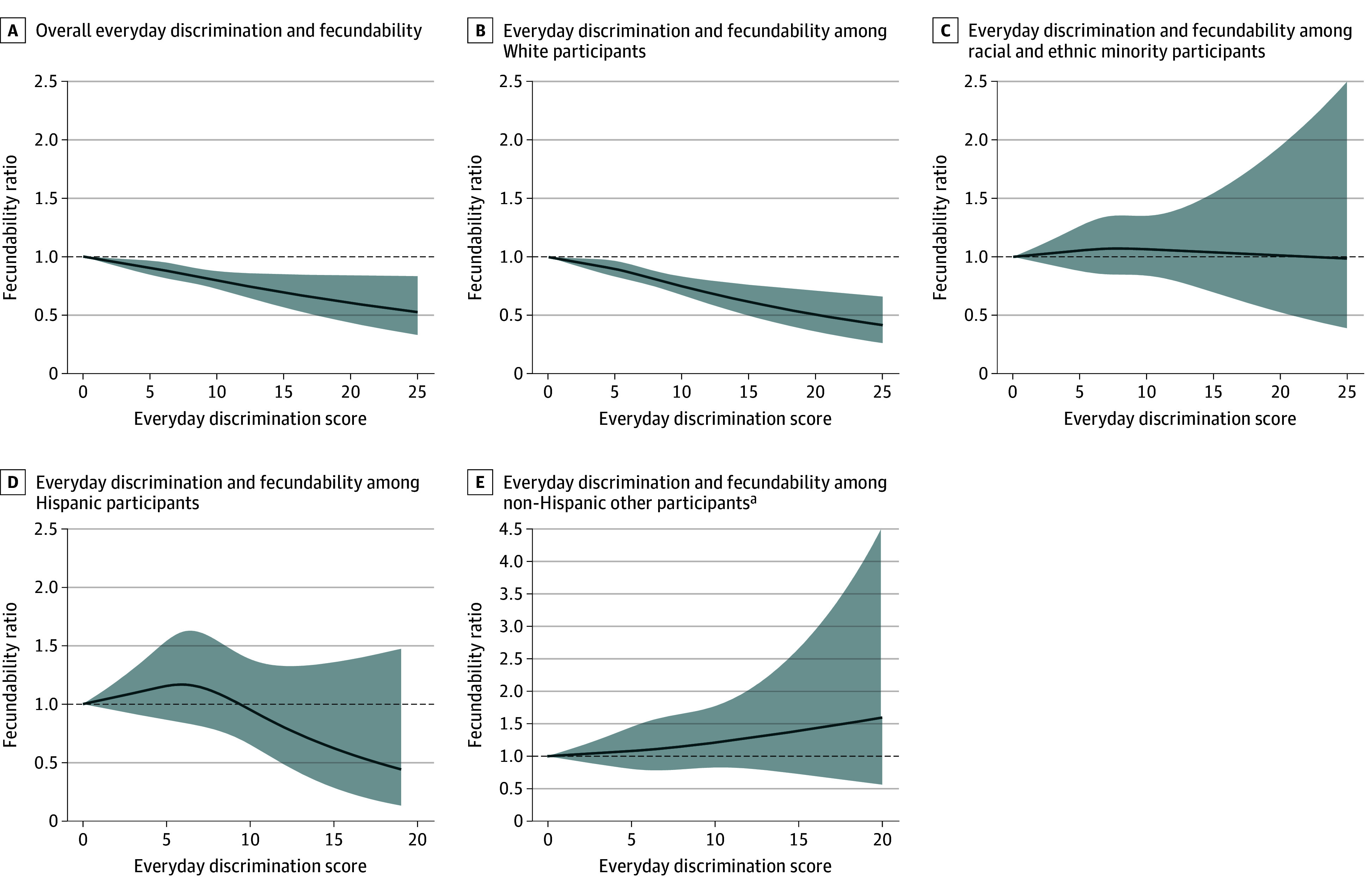
Restricted Cubic Splines of the Everyday Discrimination Summary Variable and Fecundability The graphs include 3 knots located at the 50th, 75th, and 95th percentiles. The reference level is the minimum value of the exposure (score = 0). Blue solid lines indicate the fecundability ratio; shading indicates 95% CIs. ^a^Includes American Indian or Alaska Native, Asian, Native Hawaiian or Other Pacific Islander, and multiracial or other race.

Everyday discrimination was associated with lower fecundability. Associations were similar in minimally and fully adjusted models (eTable 5 in Supplement 1). We henceforth report fully adjusted FRs (Table 2 and Figure 1). The FRs ranged from 0.74 to 0.85 for different forms of everyday discrimination (a few times or more per month vs none for specific type), including feeling disrespected (FR, 0.80 [95% CI, 0.71-0.89]), feeling that they had received poor service (FR, 0.81, [95% CI, 0.63-1.04]), believing others acted as if they were not smart (FR, 0.85 [95% CI, 0.76-0.94]), feeling that others were afraid of them (FR, 0.84 [95% CI, 0.67-1.05]), and feeling harassed (FR, 0.74 [95% CI, 0.64-0.87]). Higher scores of everyday discrimination were also associated with reduced fecundability (5-6 vs 0: FR, 0.91 [95% CI, 0.83-0.99]; ≥7 vs 0: FR, 0.82 [95% CI, 0.75-0.90]). When stratified by race and ethnicity, reduced fecundability was mostly observed among White participants who experienced everyday discrimination (FR, 0.79 [95% CI, 0.71-0.87]).

**Table 2.** Fecundability and Everyday and Lifetime Discrimination, Overall and Stratified by Race and Ethnicity^a^

Discrimination experience	Overall (N = 6578)				White participants (n = 5701)		Racial and ethnic minority participants (n = 877)^b^	
	No. of cycles	No. of pregnancies	Adjusted FR (95% CI)		No. of pregnancies	Fully adjusted FR (95% CI)^d^	No. of pregnancies	Fully adjusted FR (95% CI)^d^
			Minimally^c^	Fully^d^				
**Everyday **								
Type of experience^e^								
Disrespect								
Never	9615	1681	1 [Reference]	1 [Reference]	1532	1 [Reference]	149	1 [Reference]
<Once/y	8163	1330	0.96 (0.89-1.02)	0.97 (0.91-1.03)	1172	0.96 (0.90-1.03)	158	1.02 (0.82-1.26)
A few times/y	7323	1142	0.91 (0.85-0.97)	0.93 (0.87-1.00)	952	0.92 (0.85-0.99)	190	1.01 (0.83-1.24)
≥A few times/mo	2715	348	0.76 (0.69-0.85)	0.80 (0.71-0.89)	290	0.77 (0.68-0.87)	58	0.96 (0.72-1.28)
Poor service								
Never	17 866	3028	1 [Reference]	1 [Reference]	2763	1 [Reference]	265	1 [Reference]
<Once/y	6573	1002	0.93 (0.87-0.99)	0.95 (0.89-1.01)	837	0.93 (0.87-1.00)	165	1.07 (0.89-1.28)
A few times/y	2940	419	0.87 (0.79-0.96)	0.89 (0.81-0.98)	310	0.86 (0.77-0.96)	109	1.06 (0.86-1.30)
≥A few times/mo	437	52	0.76 (0.59-0.98)	0.81 (0.63-1.04)	36	0.70 (0.51-0.96)	16	1.25 (0.80-1.97)
Not smart								
Never	9658	1646	1 [Reference]	1 [Reference]	1462	1 [Reference]	184	1 [Reference]
<Once/y	6923	1154	0.99 (0.93-1.06)	1.00 (0.93-1.07)	1021	0.99 (0.92-1.07)	133	1.06 (0.86-1.30)
A few times/y	8427	1323	0.93 (0.87-0.99)	0.95 (0.89-1.01)	1151	0.93 (0.87-1.00)	172	1.03 (0.85-1.26)
≥A few times/mo	2808	378	0.81 (0.73-0.90)	0.85 (0.76-0.94)	312	0.83 (0.74-0.93)	66	0.96 (0.73-1.27)
Afraid of you								
Never	23 222	3873	1 [Reference]	1 [Reference]	3437	1 [Reference]	436	1 [Reference]
<Once/y	2579	368	0.90 (0.81-0.99)	0.91 (0.83-1.01)	303	0.89 (0.80-0.99)	65	1.04 (0.81-1.33)
A few times/y	1505	194	0.81 (0.71-0.93)	0.82 (0.72-0.94)	156	0.80 (0.69-0.93)	38	0.98 (0.71-1.35)
≥A few times/mo	510	66	0.80 (0.64-1.01)	0.84 (0.67-1.05)	50	0.79 (0.61-1.02)	16	1.07 (0.67-1.71)
Harassed								
Never	12 585	2132	1 [Reference]	1 [Reference]	1899	1 [Reference]	233	1 [Reference]
<Once/y	8944	1443	0.96 (0.91-1.02)	0.97 (0.92-1.04)	1248	0.96 (0.90-1.02)	195	1.10 (0.92-1.31)
A few times/y	4852	761	0.93 (0.87-1.01)	0.96 (0.89-1.03)	666	0.95 (0.88-1.03)	95	1.03 (0.82-1.29)
≥A few times/mo	1435	165	0.71 (0.61-0.83)	0.74 (0.64-0.87)	133	0.69 (0.59-0.82)	32	1.03 (0.72-1.48)
Summary variable (score)^f^								
None (0)	4733	845	1 [Reference]	1 [Reference]	774	1 [Reference]	71	1 [Reference]
Low (1-2)	5475	969	1.00 (0.92-1.09)	1.00 (0.92-1.09)	879	1.01 (0.93-1.11)	90	1.00 (0.74-1.34)
Medium (3-4)	6807	1133	0.96 (0.89-1.04)	0.97 (0.90-1.06)	1009	0.98 (0.90-1.07)	124	0.96 (0.72-1.26)
High (5-6)	5456	836	0.89 (0.81-0.97)	0.91 (0.83-0.99)	708	0.88 (0.80-0.97)	128	1.13 (0.86-1.49)
Very high (≥7)	5345	718	0.79 (0.72-0.87)	0.82 (0.75-0.90)	576	0.79 (0.71-0.87)	142	1.02 (0.77-1.35)
**Lifetime **								
Any experience								
No	14 140	2436	1 [Reference]	1 [Reference]	2195	1 [Reference]	241	1 [Reference]
Yes	13 676	2065	0.91 (0.86-0.96)	0.93 (0.88-0.99)	1751	0.95 (0.89-1.01)	314	0.80 (0.68-0.94)
Type of experience^e^^,^^g^								
On the job	9874	1469	0.91 (0.86-0.97)	0.94 (0.88-0.99)	1249	0.95 (0.89-1.01)	220	0.84 (0.72-0.99)
In housing	1911	269	0.92 (0.82-1.03)	0.95 (0.85-1.07)	214	0.94 (0.83-1.07)	55	0.93 (0.72-1.22)
By police	1770	264	0.97 (0.86-1.09)	1.01 (0.89-1.13)	193	1.03 (0.90-1.18)	71	0.95 (0.74-1.20)
In the courts	780	111	0.95 (0.79-1.13)	0.99 (0.83-1.19)	85	1.01 (0.83-1.24)	26	0.94 (0.64-1.38)
At school	6358	962	0.94 (0.88-1.00)	0.97 (0.91-1.04)	779	0.98 (0.91-1.05)	183	0.92 (0.77-1.09)
Getting medical care	4338	603	0.85 (0.79-0.92)	0.88 (0.81-0.95)	508	0.88 (0.81-0.96)	95	0.83 (0.67-1.03)
No. of experiences^h^								
0	14 140	2436	1 [Reference]	1 [Reference]	2195	1 [Reference]	241	1 [Reference]
1	6782	1061	0.93 (0.87-0.99)	0.94 (0.88-1.01)	931	0.96 (0.89-1.03)	130	0.84 (0.69-1.03)
2	3901	580	0.89 (0.82-0.97)	0.92 (0.85-1.00)	489	0.95 (0.87-1.05)	91	0.71 (0.57-0.89)
≥3	2993	424	0.87 (0.79-0.95)	0.91 (0.82-1.01)	331	0.92 (0.82-1.02)	93	0.84 (0.66-1.06)

Abbreviation: FR, fecundability ratio.

^a^ Data are from the Pregnancy Study Online cohort that includes female participants, aged 21 to 45 years, in the US and Canada who were recruited online from June 2013 to January 2023.

^b^ Includes non-Hispanic Black, Hispanic, and non-Hispanic other race (including American Indian or Alaskan Native, Asian, Native Hawaiian or Other Pacific Islander, and multiracial or other race).

^c^ Adjusted for age and race and ethnicity.

^d^ Adjusted for age, parental educational level, adverse childhood experiences, childhood financial hardship, and race and ethnicity (with the exception of stratified models).

^e^ Not mutually exclusive.

^f^ Includes disrespect, poor service, not smart, afraid of you, or harassed and was created after assigning a score to each Likert scale and summing type of everyday discrimination (ranging from 0 to 25, in which 0 = never, 1 = less than once a year, 2 = a few times a year, 3 = a few times a month, 4 = at least once a week, and 5 = almost every day). The score variable was then divided into 5 categories based on the cohort distribution: none (0 [reference category]), low (1-2), medium (3-4), high (5-6), and very high (≥7).

^g^ Exposure referent = none to that type of discrimination.

^h^ Scores range from 0 to 3 or more, with 3 or more indicating greater lifetime discrimination.

### Lifetime Discrimination and Fecundability

Any experience of lifetime discrimination (vs none) was associated with lower fecundability (FR, 0.93 [95% CI, 0.88-0.99]) (Table 2). Associations were observed among those who reported discrimination when receiving medical care (FR, 0.88 [95% CI, 0.81-0.95]) or on the job (FR, 0.94 [95% CI, 0.88-0.99]). We observed an FR of 0.91 (95% CI 0.82-1.01) for lifetime discrimination and fecundability (≥3 events vs none). Associations were mostly observed among racial and ethnic minority participants compared with White participants for any lifetime (FR, 0.80 [95% CI, 0.68-0.94] compared with FR, 0.95 [95% CI, 0.89-1.01]), job-related (FR, 0.84 [95% CI, 0.72-0.99] compared with FR 0.95 [95% CI, 0.89-1.01]), and police-related (FR, 0.95 [95% CI, 0.74-1.20] compared with FR 1.03 [95% CI, 0.90-1.18]) discrimination (Table 2).

### Attribution to Everyday and Lifetime Discrimination and Fecundability

**Figure 2.**
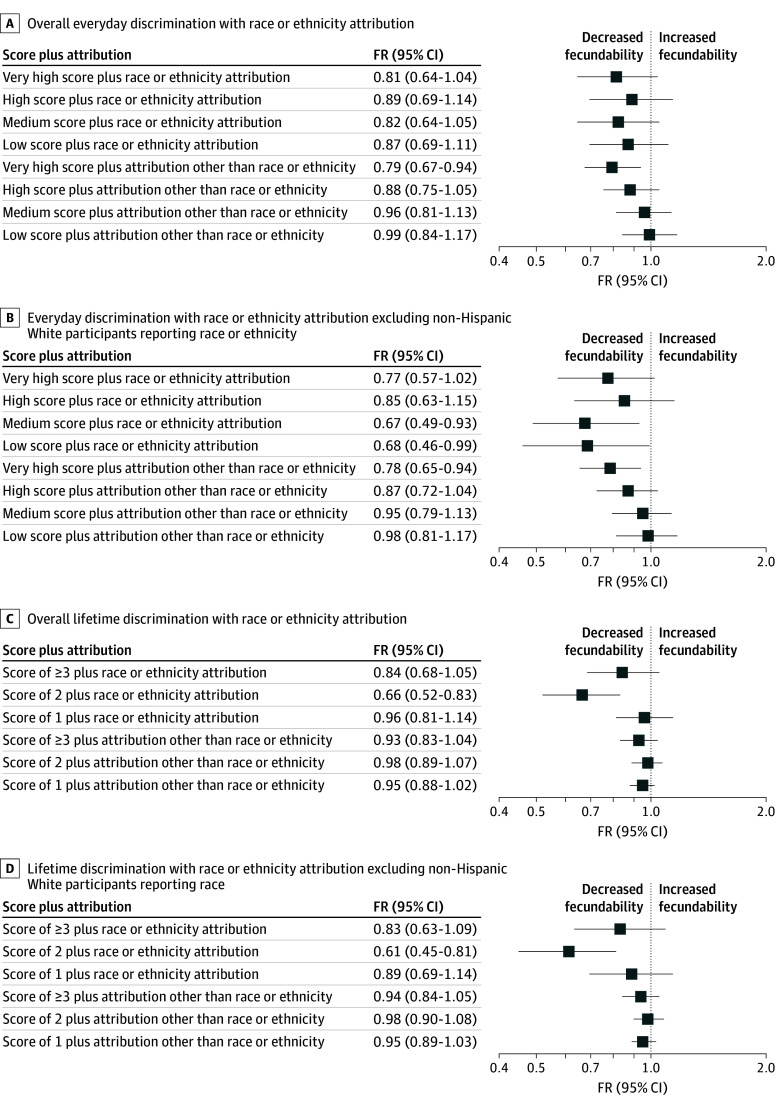
Forest Plots of Fecundability and Everyday and Lifetime Discrimination With Race or Ethnicity Attribution A and B, Excludes participants who reported ever experiencing everyday discrimination and did not report an attribution when modeled in regression analyses. C and D, Excludes participants who reported ever experiencing lifetime discrimination and did not report an attribution when modeled in regression analyses. FR indicates fecundability ratio.

Fecundability was generally lower among participants who experienced everyday discrimination and attributed their experiences to race or ethnicity compared with participants reporting no everyday discrimination (Figure 2 and eTable 6 in Supplement 1). However, reporting everyday discrimination scores of 7 or more with attributions other than race or ethnicity was associated with reduced fecundability. After excluding White participants who reported race or ethnicity as an attribution, low and medium everyday discrimination scores were associated with fecundability when race or ethnicity was the reported attribution. Associations were observed among participants who experienced 2 events of lifetime discrimination and attributed them to race or ethnicity.

Fecundability was lowest among White participants who experienced everyday discrimination scores of 7 or more and attribution other than sex or gender compared with no everyday discrimination (eTable 7 in Supplement 1). Fecundability was reduced among racial and ethnic minority participants who experienced 2 events of lifetime discrimination, regardless of whether they attributed their experience to sex or gender.

### Responses to Everyday and Lifetime Discrimination and Fecundability

**Figure 3.**
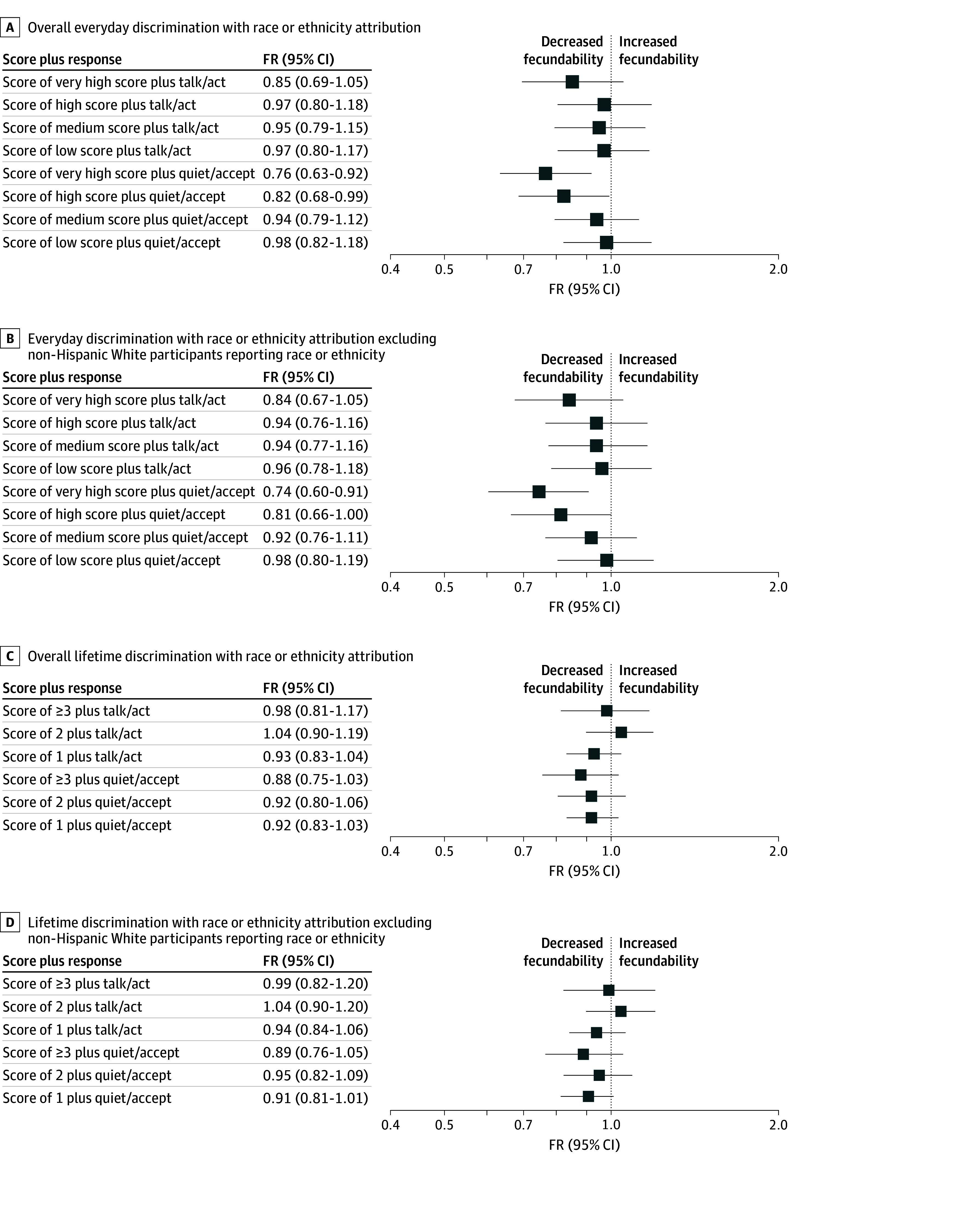
Forest Plots of Fecundability and Everyday and Lifetime Discrimination With Response Types A and B, Excludes participants who reported ever experiencing everyday discrimination and had a moderately involved response to discrimination. C and D, Excludes participants who reported ever experiencing lifetime discrimination and had a moderately involved response to discrimination when modeled in regression analyses. FR indicates fecundability ratio.

Compared with no everyday discrimination, fecundability was lowest among those with everyday discrimination scores of 7 or more who tended to keep quiet and accept their experiences (FR, 0.76 [95% CI, 0.63-0.92]) (Figure 3 and eTable 8 in Supplement 1). Similarly, those who experienced 3 or more lifetime discrimination events with a quiet and accept response had the lowest fecundability compared with no lifetime discrimination (FR, 0.88 [95% CI, 0.75-1.03]). The data were similar after excluding White participants reporting race or ethnicity as an attribution.

### Sensitivity Analyses

Associations between experiencing everyday and lifetime discrimination and fecundability were generally mostly observed among participants who completed the LCEQ retrospectively vs prospectively (eTable 9 in Supplement 1) and among those with longer pregnancy-attempt times at enrollment (eTable 10 in Supplement 1). Stratified analyses by country of residence and by calendar period showed similar results to the main analyses (eTables 11-13 in Supplement 1).

## Discussion

### Main Findings

In this preconception cohort study, experiences of everyday and lifetime discrimination were associated with reduced fecundability. Everyday discrimination was associated with decreased fecundability among White participants, especially those reporting greater exposure to harassment. Conversely, associations with lifetime discrimination, especially job-related and medical-related discrimination, were mostly observed among racial and ethnic minority participants. Participants who attributed their discrimination to race or ethnicity and those who kept quiet and accepted their experiences were more likely to have reduced fecundability.

### Comparison With Other Findings

To our knowledge, there are no studies of interpersonal discrimination and fecundability. Previous studies have focused on racial disparities in infertility, access to infertility treatment, and treatment success rates.^3,4,56,57,58,59,60^ Most but not all^56,60^ studies reported a higher prevalence of infertility among Black women compared with Hispanic or White women in the US,^3,4,5,57,58,59^ including data from our team’s cohort.^6^ We observed a larger proportion of higher levels of everyday and lifetime discrimination among racial and ethnic minority participants compared with White participants, similar to findings from previous literature.^32,61,62,63^

### Interpretation

While our study does not examine causal mechanisms, discrimination may lead to reduced fecundability with multiple pathways, such as reduced access to and poor quality of medical services, including reproductive services.^17,24^ Discrimination can also contribute to weathering, which refers to the allostatic load of cumulative stress over the life course.^5,11,17,25,26^ The weathering hypothesis states that individuals experience heightened vulnerability to premature health deterioration as a result of persistent, high-effort coping with discrimination-associated stress.^64,65^

The lifetime discrimination subtype mostly observed with reduced fecundability was medical care discrimination. Discrimination in medical care can result in dismissal of patients’ concerns, distrust of the medical system, and delayed diagnosis of health conditions that may cause infertility.^5,15,17,19,26^ Due to systemic racism, racial and ethnic minority groups are also more likely to live in residential areas where good quality medical care is lacking.^5,17,19,25^ These factors may explain why we observed the lowest fecundability among participants who experienced high levels of discrimination and attributed the discrimination to race or ethnicity.

Among racial and ethnic minority participants, job discrimination was associated with reduced fecundability. Workplace discrimination may lead to early job loss, which may, in turn, increase financial hardship.^26^ Financial hardship is directly associated with lower fecundability^66^ and is also a reason for delayed childbearing or advanced maternal age, which are risk factors for reduced fecundability.^35,59,67^ Despite controlling for age in our analyses, we observed lower fecundability for job discrimination.

We observed lower fecundability among White participants who reported higher levels of everyday discrimination, especially those who reported frequent harassment. This was not observed among racial and ethnic minority participants, but heterogeneity of individuals within this category could have attenuated results. Previous studies have observed associations between everyday discrimination and adverse health outcomes for White compared with Black individuals.^11,31^ Although Black individuals experience higher levels of adversity, their perception and appraisal of their stressfulness are likely conditioned by earlier and more frequent exposure to discrimination.^11,31^ Black individuals may therefore respond with greater flexibility and preparedness and have better social support, which could attenuate associations.^11,31^ We observed that participants who kept quiet and accepted their experiences were more likely to have reduced fecundability. Active coping strategies, including confrontation and seeking social support, may partly shield individuals from discrimination-related distress.^68,69^ However, maladaptive coping strategies such as self-silencing or emotional suppression may result in negative health outcomes. More research is needed to clarify the pathways by which discrimination may influence fecundability.

### Implications

Our findings have substantial implications for individuals who are trying to conceive, as well as for public health. Discrimination may exacerbate existing disparities in reproductive health by adding stress and uncertainty to the family planning process. The association between everyday discrimination, especially harassment, and fecundability among White individuals pinpoints the potential role of acute stressors in fertility. Meanwhile, the associations of lifetime job-related and medical-related discrimination with fecundability among racial and ethnic minority individuals underscore the lasting impact of structural inequities in employment and health care access. These findings call for greater awareness among clinicians and policymakers and the need to address racism and bias in health care, workplace policies, and society to help ensure reproductive equity and possibly improve fertility outcomes.

### Strengths and Limitations

Strengths of this study include the large sample of participants along the full fertility spectrum. Given the lack of studies on discrimination and fecundability in North America, particularly in Canada, our study provides novel data. We enrolled participants preconceptionally and restricted analyses to less than 6 cycles of pregnancy-attempt time at enrollment, which may have reduced biases due to left truncation, selection, and reverse causation. Most pregnancies were confirmed using home pregnancy tests, thereby reducing outcome misclassification. We assessed discrimination using widely used, validated measures,^33,70^ and the prospective design may have minimized the potential for differential exposure misclassification.

Study limitations include the underrepresentation of low-income and racial and ethnic minority groups relative to the general population of reproductive-aged females,^71,72^ which may have reduced generalizability. Heterogeneity within the racial and ethnic minority group may have also obscured associations. We cannot rule out the potential for recall bias among participants who completed the LCEQ retrospectively. Moreover, selection bias was possible given that we observed longer pregnancy-attempt times at enrollment among those experiencing greater discrimination.

## Conclusions

The findings of this cohort study suggest that experiences of everyday and lifetime discrimination were associated with lower fecundability. Associations varied by frequency and type of discrimination as well as attribution and response type. More research is needed to address discrimination in society and its association with fertility.

## Notes

Supplement 1. **eFigure.** Directed Acyclic Graph of the Hypothesized Association Between Discrimination and Fecundability **eTable 1.** Distribution of Everyday and Lifetime Discrimination (PRESTO 2013-2023) **eTable 2.** Distribution of Attribution and Response Types to Discrimination (PRESTO 2013-2023) **eTable 3.** Characteristics of 13 394 Participants by Completion of the Supplemental Life Course Experiences Questionnaire (PRESTO 2013-2023) **eTable 4.** Characteristics of 6578 Participants by Loss to Follow-Up and Timing of Completion for the Supplemental Life Course Experiences Questionnaire (PRESTO 2013-2023) **eTable 5.** Unadjusted and Adjusted Fecundability Ratios in Relation to Everyday and Lifetime Discrimination (PRESTO 2013-2023) **eTable 6.** Fecundability in Relation to Everyday and Lifetime Discrimination With “Race or Ethnicity” Attribution (PRESTO 2013-2023) **eTable 7.** Fecundability in Relation to Everyday and Lifetime Discrimination With “Sex or Gender” Attribution (PRESTO 2013-2023) **eTable 8.** Fecundability in Relation to Everyday and Lifetime Discrimination With Response Types (PRESTO 2013-2023) **eTable 9.** Fecundability in Relation to Everyday and Lifetime Discrimination by Timing of LCEQ Completion (PRESTO 2013-2023) **eTable 10.** Fecundability in Relation to Discrimination by Pregnancy Attempt Time at Enrollment (PRESTO 2013-2023) **eTable 11.** Fecundability in Relation to Discrimination by Country of Residence at Enrollment (PRESTO 2013-2023) **eTable 12.** Fecundability in Relation to Discrimination by June 2020 for LCEQ Completion (PRESTO 2013-2023) **eTable 13.** Fecundability in Relation to Discrimination by June 2020 for LCEQ Completion and Race or Ethnicity (PRESTO 2013-2023)

Supplement 2.
Data Sharing Statement
