# Supplementary material for: Fecundability in Association With Everyday and Lifetime Discrimination
Source: JAMA Netw Open. 2025 Jul 14;8(7):e2520597. doi: 10.1001/jamanetworkopen.2025.20597 (PMC12260993; doi:10.1001/jamanetworkopen.2025.20597)
Supplement: Supplement 1. — eFigure. Directed Acyclic Graph of the Hypothesized Association Between Discrimination and Fecundability eTable 1. Distribution of Everyday and Lifetime Discrimination (PRESTO 2013-2023) eTable 2. Distribution of Attribution and Response Types to Discrimination (PRESTO 2013-2023) eTable 3. Characteristics of 13 394 Participants by Completion of the Supplemental Life Course Experiences Questionnaire (PRESTO 2013-2023) eTable 4. Characteristics of 6578 Participants by Loss to Follow-Up and Timing of Completion for the Supplemental Life Course Experiences Questionnaire (PRESTO 2013-2023) eTable 5. Unadjusted and Adjusted Fecundability Ratios in Relation to Everyday and Lifetime Discrimination (PRESTO 2013-2023) eTable 6. Fecundability in Relation to Everyday and Lifetime Discrimination With “Race or Ethnicity” Attribution (PRESTO 2013-2023) eTable 7. Fecundability in Relation to Everyday and Lifetime Discrimination With “Sex or Gender” Attribution (PRESTO 2013-2023) eTable 8. Fecundability in Relation to Everyday and Lifetime Discrimination With Response Types (PRESTO 2013-2023) eTable 9. Fecundability in Relation to Everyday and Lifetime Discrimination by Timing of LCEQ Completion (PRESTO 2013-2023) eTable 10. Fecundability in Relation to Discrimination by Pregnancy Attempt Time at Enrollment (PRESTO 2013-2023) eTable 11. Fecundability in Relation to Discrimination by Country of Residence at Enrollment (PRESTO 2013-2023) eTable 12. Fecundability in Relation to Discrimination by June 2020 for LCEQ Completion (PRESTO 2013-2023) eTable 13. Fecundability in Relation to Discrimination by June 2020 for LCEQ Completion and Race or Ethnicity (PRESTO 2013-2023) [file jamanetwopen-e2520597-s001.pdf]

Supplemental Online Content

Ukah UV, Lovett SM, Boynton-Jarrett R, et al. Fecundability in association with everyday and lifetime discrimination. *JAMA Netw Open*. 2025;8(7):e2520597. doi:10.1001/jamanetworkopen.2025.20597

eFigure. Directed Acyclic Graph of the Hypothesized Association Between Discrimination and Fecundability
eTable 1. Distribution of Everyday and Lifetime Discrimination (PRESTO 2013-2023)
eTable 2. Distribution of Attribution and Response Types to Discrimination (PRESTO 2013-2023)
eTable 3. Characteristics of 13 394 Participants by Completion of the Supplemental Life Course Experiences Questionnaire (PRESTO 2013-2023)
eTable 4. Characteristics of 6578 Participants by Loss to Follow-Up and Timing of Completion for the Supplemental Life Course Experiences Questionnaire (PRESTO 2013-2023)
eTable 5. Unadjusted and Adjusted Fecundability Ratios in Relation to Everyday and Lifetime Discrimination (PRESTO 2013-2023)
eTable 6. Fecundability in Relation to Everyday and Lifetime Discrimination With "Race or Ethnicity" Attribution (PRESTO 2013-2023)
eTable 7. Fecundability in Relation to Everyday and Lifetime Discrimination With "Sex or Gender" Attribution (PRESTO 2013-2023)
eTable 8. Fecundability in Relation to Everyday and Lifetime Discrimination With Response Types (PRESTO 2013-2023)
eTable 9. Fecundability in Relation to Everyday and Lifetime Discrimination by Timing of LCEQ Completion (PRESTO 2013-2023)
eTable 10. Fecundability in Relation to Discrimination by Pregnancy Attempt Time at Enrollment (PRESTO 2013-2023)
eTable 11. Fecundability in Relation to Discrimination by Country of Residence at Enrollment (PRESTO 2013-2023)
eTable 12. Fecundability in Relation to Discrimination by June 2020 for LCEQ Completion (PRESTO 2013-2023)
eTable 13. Fecundability in Relation to Discrimination by June 2020 for LCEQ Completion and Race or Ethnicity (PRESTO 2013-2023)

This supplemental material has been provided by the authors to give readers additional information about their work.

© 2025 Ukah UV et al. *JAMA Network Open*.

eFigure. Directed Acyclic Graph of the Hypothesized Association Between Discrimination and Fecundability

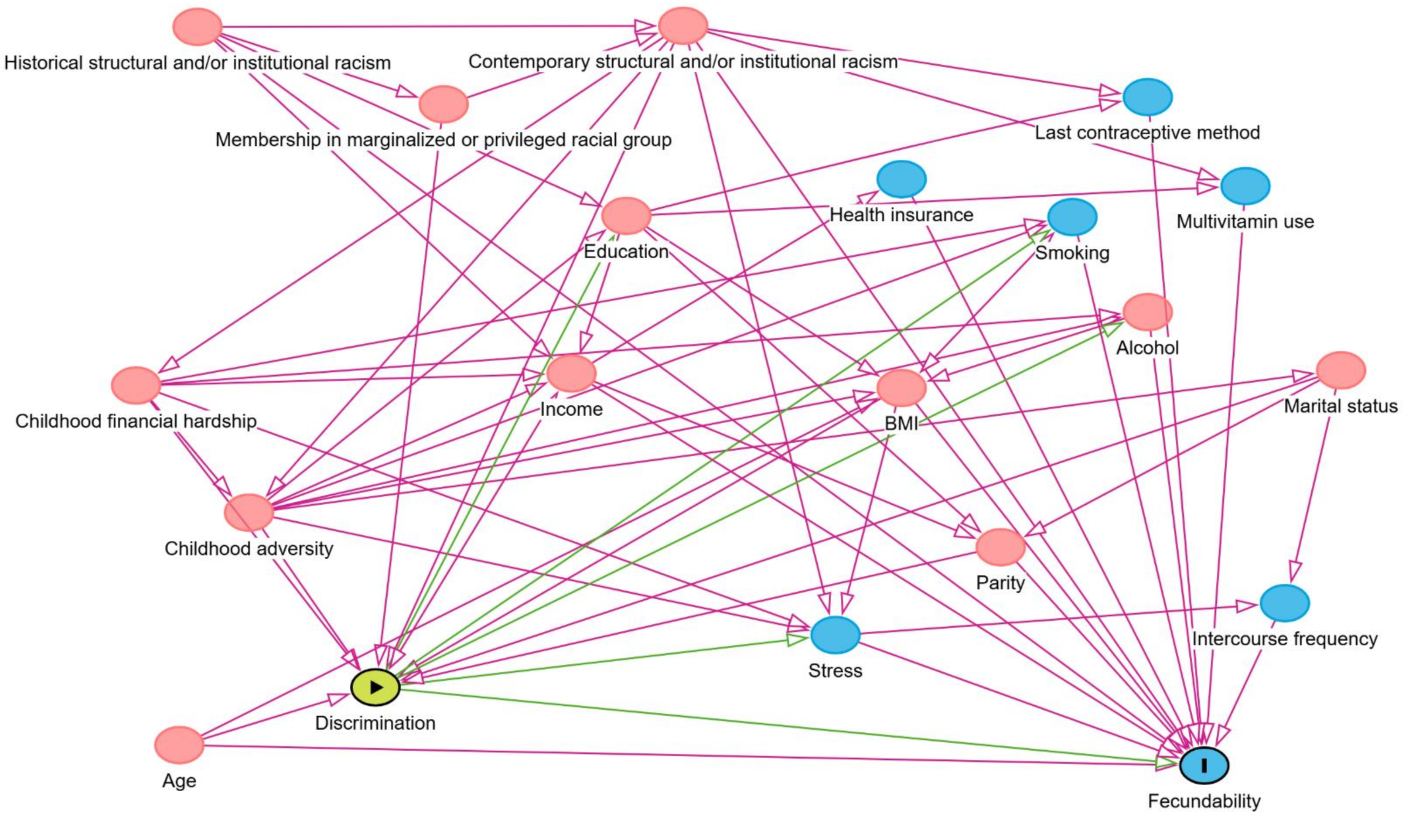

Note: This directed acyclic graph was informed in part by Howe CJ, Bailey ZD, Raifman JR, & Jackson JW. Recommendations for using causal diagrams to study racial health disparities. Am J Epidemiol. 2022; 191(12):1981-1989. doi: 10.1093/aje/kwac140. Examples of historical structural and/or institutional racism include racist policies (*e.g.,* slavery, Jim Crow laws); Examples of contemporary structural and/or institutional racism (*e.g.,* discriminatory mortgage lending practices, medical care, and job hiring); and "Membership in marginalized or privileged racial group" was modeled in regression analyses using a variable on self-identified race/ethnicity.

© 2025 Ukah UV et al. *JAMA Network Open*.

eTable 1. Distribution of Everyday and Lifetime Discrimination (PRESTO 2013-2023)

"Before you enrolled in the study…"	Overall	Hispanic	Non-Hispanic Black	Non-Hispanic White	Non-Hispanic Other[a]	BIPOC[b]
	(n=6,578)	(n=375)	(n=110)	(n=5,701)	(n=392)	(n=877)
**EVERYDAY EXPERIENCES**						
"In your day-to-day life, how often did any of the following things happen to you?"						
Disrespect ("You were treated with less courtesy or respect than other people?"), %						
Never	2,350 (35.7)	118 (31.5)	18 (16.4)	2,113 (37.1)	101 (25.8)	237 (27.0)
Less than once a year	1,941 (29.5)	103 (27.5)	22 (20.0)	1,692 (29.7)	124 (31.6)	249 (28.4)
A few times a year	1,698 (25.8)	114 (30.4)	54 (49.1)	1,405 (24.6)	125 (31.9)	293 (33.4)
A few times a month	338 (5.1)	23 (6.1)	3 (2.7)	288 (5.1)	24 (6.1)	50 (5.7)
At least once a week	165 (2.5)	12 (3.2)	7 (6.4)	131 (2.3)	15 (3.8)	34 (3.9)
Almost every day	86 (1.3)	5 (1.3)	6 (5.5)	72 (1.3)	3 (0.8)	14 (1.6)
Poor service ("You received poorer service than other people at restaurants or stores?"), %						
Never	4,297 (65.3)	205 (54.7)	23 (20.9)	3,877 (68.0)	192 (49.0)	420 (47.9)
Less than once a year	1,526 (23.2)	97 (25.9)	33 (30.0)	1,270 (22.3)	126 (32.1)	256 (29.2)
A few times a year	659 (10.0)	64 (17.1)	44 (40.0)	484 (8.5)	67 (17.1)	175 (20.0)
A few times a month	75 (1.1)	7 (1.9)	8 (7.3)	54 (1.0)	6 (1.5)	21 (2.4)
At least once a week	16 (0.2)	1 (0.3)	2 (1.8)	12 (0.2)	1 (0.3)	4 (0.5)
Almost every day	5 (0.1)	1 (0.3)	0 (0.0)	4 (0.1)	0 (0.0)	1 (0.1)
Not smart ("People acted as if they thought you were not smart?"), %						
Never	2,324 (35.3)	124 (33.1)	21 (19.1)	2,033 (35.7)	146 (37.2)	291 (33.2)
Less than once a year	1,663 (25.3)	78 (20.8)	29 (26.4)	1,463 (25.7)	93 (23.7)	200 (22.8)
A few times a year	1,970 (29.9)	132 (35.2)	35 (31.8)	1,694 (29.7)	109 (27.8)	276 (31.5)
A few times a month	434 (6.6)	31 (8.3)	15 (13.6)	362 (6.4)	26 (6.6)	72 (8.2)
At least once a week	142 (2.2)	7 (1.9)	6 (5.5)	117 (2.1)	12 (3.1)	25 (2.9)
Almost every day	45 (0.7)	3 (0.8)	4 (3.6)	32 (0.6)	6 (1.5)	13 (1.5)
Afraid of you ("People acted as if they were afraid of you?"), %						
Never	5,575 (84.8)	300 (80.0)	59 (53.6)	4,903 (86.0)	313 (79.9)	672 (76.6)
Less than once a year	571 (8.7)	39 (10.4)	23 (20.9)	462 (8.1)	47 (12.0)	109 (12.4)
A few times a year	322 (4.9)	27 (7.2)	20 (18.2)	253 (4.4)	22 (5.6)	69 (7.9)
A few times a month	80 (1.2)	6 (1.6)	4 (3.6)	64 (1.1)	6 (1.5)	16 (1.8)
At least once a week	21 (0.3)	3 (0.8)	3 (2.7)	11 (0.2)	4 (1.0)	10 (1.1)
Almost every day	9 (0.1)	0 (0.0)	1 (0.9)	8 (0.1)	0 (0.0)	1 (0.1)
Harassed ("You were threatened or harassed?"), %						
Never	3,047 (46.3)	176 (46.9)	46 (41.8)	2,659 (46.6)	166 (42.4)	388 (44.2)
Less than once a year	2,096 (31.9)	113 (30.1)	49 (44.6)	1,807 (31.7)	127 (32.4)	289 (33.0)
A few times a year	1,152 (17.5)	68 (18.1)	11 (10.0)	999 (17.5)	74 (18.9)	153 (17.5)
A few times a month	190 (2.9)	14 (3.7)	2 (1.8)	155 (2.7)	19 (4.9)	35 (4.0)
At least once a week	66 (1.0)	2 (0.5)	2 (1.8)	58 (1.0)	4 (1.0)	8 (0.9)
Almost every day	27 (0.4)	2 (0.5)	0 (0.0)	23 (0.4)	2 (0.5)	4 (0.5)
**LIFETIME EXPERIENCES**						
"Have you ever been treated unfairly in any of the following circumstances?"						
"Job (hiring, promotion, firing)", %	2,274 (34.6)	146 (38.9)	55 (50.0)	1,902 (33.4)	171 (43.6)	372 (42.4)
"Housing (renting, buying, mortgage)", %	432 (6.6)	34 (9.1)	21 (19.1)	338 (5.9)	39 (10.0)	94 (10.7)
"Police (stopped, searched, threatened)", %	419 (6.4)	49 (13.1)	36 (32.7)	288 (5.1)	46 (11.7)	131 (14.9)
"In the courts", %	183 (2.8)	18 (4.8)	8 (7.3)	137 (2.4)	20 (5.1)	46 (5.3)
"At school", %	1,469 (22.3)	113 (30.1)	54 (49.1)	1,166 (20.5)	136 (34.7)	303 (34.6)
"Getting medical care", %	971 (14.8)	70 (18.7)	32 (29.1)	801 (14.1)	68 (17.4)	170 (19.4)

© 2025 Ukah UV et al. *JAMA Network Open*.

Note: PRESTO = Pregnancy Study Online; [a]Includes Asian or Pacific Islander, American Indian or Alaskan Native, multiple races, or some other race; [b]Includes non-Hispanic Black, Hispanic, non-Hispanic Other (defined as participant self-identified as Asian or Pacific Islander, American Indian or Alaskan Native, multiple races, or some other race)

© 2025 Ukah UV et al. *JAMA Network Open*.

eTable 2. Distribution of Attribution and Response Types to Discrimination (PRESTO 2013-2023)

"Before you enrolled in the study…"	Overall	Hispanic	Non-Hispanic Black	Non-Hispanic White	Non-Hispanic Other[a]	BIPOC[b]
	(n=6,578)	(n=375)	(n=110)	(n=5,701)	(n=392)	(n=877)
**TOTAL CASES REPORTING EVERYDAY OR LIFETIME DISCRIMINATION**	5,597 (85.1)	327 (87.2)	107 (97.3)	4,814 (84.4)	349 (89.0)	783 (89.3)
**MAIN REASONS FOR EXPERIENCES[c]**						
"What do you think was the main reason for these experiences? (Choose all that apply)"						
"Your race or ethnicity", %	702 (12.5)	144 (44.0)	95 (88.8)	292 (6.1)	171 (49.0)	410 (52.4)
"Your sex or gender", %	4,435 (79.2)	249 (76.2)	68 (63.6)	3,859 (80.2)	259 (74.2)	576 (73.6)
"Your education or income level", %	1,033 (18.5)	70 (21.4)	17 (15.9)	873 (18.1)	73 (20.9)	160 (20.4)
"Other", %	1,353 (24.2)	61 (18.7)	6 (5.6)	1,220 (25.3)	66 (18.9)	133 (17.0)
Number of main reasons selected, %						
0	110 (2.0)	3 (0.9)	4 (3.7)	93 (1.9)	10 (2.9)	17 (2.2)
1	3,781 (67.6)	167 (51.1)	32 (29.9)	3,432 (71.3)	150 (43.0)	349 (44.6)
2	1,396 (24.9)	114 (34.9)	59 (55.1)	1,071 (22.3)	152 (43.6)	325 (41.5)
≥3	310 (5.6)	43 (13.2)	12 (11.2)	218 (4.5)	37 (10.6)	92 (11.7)
**RESPONSE TYPES[c]**						
"When you felt you were treated unfairly, did you… (Choose one option)"						
Quiet ("Keep it to yourself?"), %	2,319 (41.4)	127 (38.8)	42 (39.3)	2,017 (41.9)	133 (38.1)	302 (38.6)
Talk ("Talk to other people about it?"), %	3,278 (58.6)	200 (61.2)	65 (60.8)	2,797 (58.1)	216 (61.9)	481 (61.4)
"Additionally, did you usually… (Choose one option)"						
Accept ("Accept it as a fact of life?"), %	3,970 (70.9)	218 (66.7)	77 (72.0)	3,424 (71.1)	251 (71.9)	546 (69.7)
Act ("Try to do something about it?"), %	1,627 (29.1)	109 (33.3)	30 (28.0)	1,390 (28.9)	98 (28.1)	237 (30.3)
**RESPONSE TYPE COMBINATIONS[c]**						
Quiet and accept, %	2,068 (37.0)	106 (32.4)	39 (36.5)	1,798 (37.4)	125 (35.8)	270 (34.5)
Talk and accept, %	1,902 (34.0)	112 (34.3)	38 (35.5)	1,626 (33.8)	126 (36.1)	276 (35.3)
Quiet and act, %	251 (4.5)	21 (6.4)	3 (2.8)	219 (4.6)	8 (2.3)	32 (4.1)
Talk and act, %	1,376 (24.6)	88 (26.9)	27 (25.2)	1,171 (24.3)	90 (25.8)	205 (26.2)

Note: PRESTO = Pregnancy Study Online; [a]Includes Asian or Pacific Islander, American Indian or Alaskan Native, multiple races, or some other race; [b]Includes non-Hispanic Black, Hispanic, non-Hispanic Other (defined as participant self-identified as Asian or Pacific Islander, American Indian or Alaskan Native, multiple races, or some other race); [c]Limited to participants that reported ever experiencing everyday or lifetime discrimination

© 2025 Ukah UV et al. *JAMA Network Open*.

eTable 3. Characteristics of 13 394 Participants by Completion of the Supplemental Life Course Experiences Questionnaire (PRESTO 2013-2023)

	**Completed LCEQ**	
	Yes	No
	6,578 (49.1%)	6,816 (50.9%)
Age (years), mean (SD)	30.5 (3.9)	29.8 (4.2)
Married, %	5,910 (89.8)	5,921 (86.9)
Race and ethnicity, %		
Hispanic	375 (5.7)	526 (7.7)
Non-Hispanic Black	110 (1.7)	280 (4.1)
Non-Hispanic White	5,701 (86.7)	5,538 (81.3)
Non-Hispanic Other[a]	392 (6.0)	472 (6.9)
Educational attainment (years), %		
≤12	184 (2.8)	485 (7.1)
13-15	903 (13.7)	1,797 (26.4)
16	2,203 (33.5)	2,309 (33.9)
≥17	3,288 (50.0)	2,225 (32.6)
Household income (U.S. dollars/year), %		
<$50,000	794 (12.1)	1,508 (22.1)
$50,000-$99,999	2,223 (33.8)	2,480 (36.4)
$100,000-$149,999	1,957 (29.8)	1,581 (23.2)
≥$150,000	1,604 (24.4)	984 (14.4)
Body mass index (kg/m^2), mean (SD)	27.1 (6.8)	28.7 (7.9)
Current alcohol intake ≥14 drinks/week, %	155 (2.4)	206 (3.0)
Current smoker, %	309 (4.7)	859 (12.6)
Age at menarche <12 years, %	1,570 (23.9)	1,748 (25.6)
Parous, %	2,112 (32.1)	2,312 (33.9)

Note: PRESTO = Pregnancy Study Online; LCEQ = supplemental Life Course Experiences Questionnaire; [a]Includes Asian or Pacific Islander, American Indian or Alaskan Native, multiple races, or some other race

© 2025 Ukah UV et al. *JAMA Network Open*.

eTable 4. Characteristics of 6578 Participants by Loss to Follow-Up and Timing of Completion for the Supplemental Life Course Experiences Questionnaire (PRESTO 2013-2023)

	**Lost to Follow-up**	**Not Lost to Follow-up**	**Retrospective Completion[a]**	**Prospective Completion[b]**
	493 (7.5%)	6,085 (92.5%)	3,299 (50.2%)	3,279 (49.8%)
Age (years), mean (SD)	30.2 (4.4)	30.6 (3.9)	30.3 (3.9)	30.8 (3.9)
Married, %	396 (80.3)	5,514 (90.6)	3,033 (91.9)	2,877 (87.7)
Race/ethnicity, %				
Hispanic	39 (7.9)	336 (5.5)	165 (5.0)	210 (6.4)
Non-Hispanic Black	13 (2.6)	97 (1.6)	45 (1.4)	65 (2.0)
Non-Hispanic White	402 (81.5)	5,299 (87.1)	2,894 (87.7)	2,807 (85.6)
Non-Hispanic Other[c]	39 (7.9)	353 (5.8)	195 (5.9)	197 (6.0)
Educational attainment (years), %				
≤12	41 (8.3)	143 (2.4)	90 (2.7)	94 (2.9)
13-15	110 (22.3)	793 (13.0)	490 (14.9)	413 (12.6)
16	172 (34.9)	2,031 (33.4)	1,146 (34.7)	1,057 (32.2)
≥17	170 (34.5)	3,118 (51.2)	1,573 (47.7)	1,715 (52.3)
Household income (U.S. dollars/year), %				
<$50,000	100 (20.3)	694 (11.4)	442 (13.4)	352 (10.7)
$50,000-$99,999	190 (38.5)	2,033 (33.4)	1,224 (37.1)	999 (30.5)
$100,000-$149,999	133 (27.0)	1,824 (30.0)	976 (29.6)	981 (29.9)
≥$150,000	70 (14.2)	1,534 (25.2)	657 (19.9)	947 (28.9)
Body mass index (kg/m^2), mean (SD)	30.4 (8.1)	26.8 (6.6)	26.8 (6.7)	27.3 (6.9)
Current alcohol intake ≥14 drinks/week, %	16 (3.2)	139 (2.3)	88 (2.7)	67 (2.0)
Current smoker, %	42 (8.5)	267 (4.4)	195 (5.9)	114 (3.5)
Age at menarche <12 years, %	149 (30.2)	1,421 (23.4)	774 (23.5)	796 (24.3)
Parous, %	170 (34.5)	1,942 (31.9)	1,090 (33.0)	1,022 (31.2)

Note: PRESTO = Pregnancy Study Online; [a]Retrospective completion of the supplemental questionnaire is defined as >60 days after enrollment; [b]Prospective completion of the supplemental questionnaire is defined as within 60 days of enrollment (allowing participants 30 days to complete it); [c]Includes Asian or Pacific Islander, American Indian or Alaskan Native, multiple races, or some other race

© 2025 Ukah UV et al. *JAMA Network Open*.

eTable 5. Unadjusted and Adjusted Fecundability Ratios in Relation to Everyday and Lifetime Discrimination (PRESTO 2013-2023)

	Overall (n=6,578)				
	**Pregnancies**	**Cycles**	**Unadjusted FR (95% CI)**	**Minimally Adjusted FR (95% CI)[a]**	**Fully Adjusted FR (95% CI)[b]**
**EVERYDAY DISCRIMINATION**					
**Type of Experience[c]**					
Disrespect					
Never	1,681	9,615	1.00 (Reference)	1.00 (Reference)	1.00 (Reference)
Less than once a year	1,330	8,163	0.94 (0.88-1.01)	0.96 (0.89-1.02)	0.97 (0.91-1.03)
A few times a year	1,142	7,323	0.90 (0.84-0.97)	0.91 (0.85-0.97)	0.93 (0.87-1.00)
≥A few times a month	348	2,715	0.76 (0.68-0.85)	0.76 (0.69-0.85)	0.80 (0.71-0.89)
Poor service					
Never	3,028	17,866	1.00 (Reference)	1.00 (Reference)	1.00 (Reference)
Less than once a year	1,002	6,573	0.92 (0.86-0.98)	0.93 (0.87-0.99)	0.95 (0.89-1.01)
A few times a year	419	2,940	0.85 (0.77-0.93)	0.87 (0.79-0.96)	0.89 (0.81-0.98)
≥A few times a month	52	437	0.74 (0.57-0.95)	0.76 (0.59-0.98)	0.81 (0.63-1.04)
Not smart					
Never	1,646	9,658	1.00 (Reference)	1.00 (Reference)	1.00 (Reference)
Less than once a year	1,154	6,923	0.98 (0.92-1.05)	0.99 (0.93-1.06)	1.00 (0.93-1.07)
A few times a year	1,323	8,427	0.93 (0.87-0.99)	0.93 (0.87-0.99)	0.95 (0.89-1.01)
≥A few times a month	378	2,808	0.81 (0.73-0.90)	0.81 (0.73-0.90)	0.85 (0.76-0.94)
Afraid of you					
Never	3,873	23,222	1.00 (Reference)	1.00 (Reference)	1.00 (Reference)
Less than once a year	368	2,579	0.88 (0.80-0.97)	0.90 (0.81-0.99)	0.91 (0.83-1.01)
A few times a year	194	1,505	0.80 (0.70-0.91)	0.81 (0.71-0.93)	0.82 (0.72-0.94)
≥A few times a month	66	510	0.80 (0.64-1.01)	0.80 (0.64-1.01)	0.84 (0.67-1.05)
Harassed					
Never	2,132	12,585	1.00 (Reference)	1.00 (Reference)	1.00 (Reference)
Less than once a year	1,443	8,944	0.96 (0.90-1.02)	0.96 (0.91-1.02)	0.97 (0.92-1.04)
A few times a year	761	4,852	0.93 (0.86-1.00)	0.93 (0.87-1.01)	0.96 (0.89-1.03)
≥A few times a month	165	1,435	0.71 (0.61-0.83)	0.71 (0.61-0.83)	0.74 (0.64-0.87)
**Summary Variable[d]**					
None (Score 0)	845	4,733	1.00 (Reference)	1.00 (Reference)	1.00 (Reference)
Low (Score 1-2)	969	5,475	0.99 (0.91-1.08)	1.00 (0.92-1.09)	1.00 (0.92-1.09)
Medium (Score 3-4)	1,133	6,807	0.95 (0.88-1.04)	0.96 (0.89-1.04)	0.97 (0.90-1.06)
High (Score 5-6)	836	5,456	0.87 (0.80-0.95)	0.89 (0.81-0.97)	0.91 (0.83-0.99)
Very High (Score ≥7)	718	5,345	0.78 (0.71-0.86)	0.79 (0.72-0.87)	0.82 (0.75-0.90)
**LIFETIME DISCRIMINATION**					
**Any Experience**					
No	2,436	14,140	1.00 (Reference)	1.00 (Reference)	1.00 (Reference)
Yes	2,065	13,676	0.89 (0.85-0.94)	0.91 (0.86-0.96)	0.93 (0.88-0.99)
**Type of Experience[c,e]**					
On the job	1,469	9,874	0.90 (0.85-0.95)	0.91 (0.86-0.97)	0.94 (0.88-0.99)
In housing	269	1,911	0.90 (0.80-1.00)	0.92 (0.82-1.03)	0.95 (0.85-1.07)
By police	264	1,770	0.93 (0.83-1.05)	0.97 (0.86-1.09)	1.01 (0.89-1.13)
In the courts	111	780	0.90 (0.76-1.08)	0.95 (0.79-1.13)	0.99 (0.83-1.19)
At school	962	6,358	0.93 (0.87-0.99)	0.94 (0.88-1.00)	0.97 (0.91-1.04)
Getting medical care	603	4,338	0.85 (0.78-0.92)	0.85 (0.79-0.92)	0.88 (0.81-0.95)
**Number of Experiences**					
0	2,436	14,140	1.00 (Reference)	1.00 (Reference)	1.00 (Reference)
1	1,061	6,782	0.92 (0.86-0.99)	0.93 (0.87-0.99)	0.94 (0.88-1.01)
2	580	3,901	0.88 (0.81-0.96)	0.89 (0.82-0.97)	0.92 (0.85-1.00)
≥3	424	2,993	0.85 (0.77-0.93)	0.87 (0.79-0.95)	0.91 (0.82-1.01)

© 2025 Ukah UV et al. *JAMA Network Open*.

Note: CI = confidence interval; FR = fecundability ratio; PRESTO = Pregnancy Study Online; [a]**Adjusted for age and race/ethnicity;** [b]**Adjusted for age, race/ethnicity, parental education, adverse childhood experiences, and childhood financial hardship;** [c]Not mutually exclusive; [d]Summary variable for everyday discrimination (disrespect, poor service, not smart, afraid of you, harassed) created after assigning a score to each Likert scale and summing across (range: 0-25, where 0 = "never," 1 = "less than once a year," 2 = "a few times a year," 3 = "a few times a month," 4 = "at least once a week," and 5 = "almost every day"); [e]Exposure referent = No to that type of discrimination

© 2025 Ukah UV et al. *JAMA Network Open*.

eTable 6. Fecundability in Relation to Everyday and Lifetime Discrimination With "Race or Ethnicity" Attribution (PRESTO 2013-2023)

	**Overall[a,b,c]**			**Excludes Non-Hispanic White participants that reported "race or ethnicity" as an attribution**		
	(n=5,597)			(n=5,305)		
	**Pregnancies**	**Cycles**	**Fully Adjusted FR (95% CI)[d]**	**Pregnancies**	**Cycles**	**Fully Adjusted FR (95% CI)[d]**
**EVERYDAY DISCRIMINATION**						
**Summary Variable x Attribution[e]**						
None (Score 0)	130	721	1.00 (Reference)	108	588	1.00 (Reference)
Low (Score 1-2) + Any attribution other than "race or ethnicity"	838	4,632	0.99 (0.84-1.17)	838	4,632	0.98 (0.81-1.17)
Medium (Score 3-4) + Any attribution other than "race or ethnicity"	1,020	5,997	0.96 (0.81-1.13)	1,020	5,997	0.95 (0.79-1.13)
High (Score 5-6) + Any attribution other than "race or ethnicity"	732	4,740	0.88 (0.75-1.05)	732	4,740	0.87 (0.72-1.04)
Very High (Score ≥7) + Any attribution other than "race or ethnicity"	586	4,314	0.79 (0.67-0.94)	586	4,314	0.78 (0.65-0.94)
Low (Score 1-2) + "Race or ethnicity" attribution	105	717	0.87 (0.69-1.11)	33	313	0.68 (0.46-0.99)
Medium (Score 3-4) + "Race or ethnicity" attribution	97	713	0.82 (0.64-1.05)	54	477	0.67 (0.49-0.93)
High (Score 5-6) + "Race or ethnicity" attribution	94	655	0.89 (0.69-1.14)	65	461	0.85 (0.63-1.15)
Very High (Score ≥7) + "Race or ethnicity" attribution	118	903	0.81 (0.64-1.04)	83	644	0.77 (0.57-1.02)
**LIFETIME DISCRIMINATION**						
**Number of Experiences x Attribution**						
0	1,721	10,128	1.00 (Reference)	1,628	9,629	1.00 (Reference)
1 + Any attribution other than "race or ethnicity"	919	5,877	0.95 (0.88-1.02)	919	5,877	0.95 (0.89-1.03)
2 + Any attribution other than "race or ethnicity"	500	3,127	0.98 (0.89-1.07)	500	3,127	0.98 (0.90-1.08)
≥3 + Any attribution other than "race or ethnicity"	329	2,244	0.93 (0.83-1.04)	329	2,244	0.94 (0.84-1.05)
1 + "Race or ethnicity" attribution	123	800	0.96 (0.81-1.14)	63	459	0.89 (0.69-1.14)
2 + "Race or ethnicity" attribution	73	720	0.66 (0.52-0.83)	50	542	0.61 (0.45-0.81)
≥3 + "Race or ethnicity" attribution	81	660	0.84 (0.68-1.05)	56	452	0.83 (0.63-1.09)

Note: CI = confidence interval; FR = fecundability ratio; PRESTO = Pregnancy Study Online; [a]Limited to participants that reported ever experiencing everyday or lifetime discrimination; [b]Excludes participants that reported ever experiencing everyday discrimination and did not report an attribution (n=101) when modeled in regression analyses; [c]Excludes participants that reported ever experiencing lifetime discrimination and did not report an attribution (n=63) when modeled in regression analyses; [d]**Adjusted for age, race/ethnicity, parental education, adverse childhood experiences, and childhood financial hardship;** [e]Summary variable for everyday discrimination (disrespect, poor service, not smart, afraid of you, harassed) created after assigning a score to each Likert scale and summing across (range: 0-25, where 0 = "never," 1 = "less than once a year," 2 = "a few times a year," 3 = "a few times a month," 4 = "at least once a week," and 5 = "almost every day")

© 2025 Ukah UV et al. *JAMA Network Open*.

eTable 7. Fecundability in Relation to Everyday and Lifetime Discrimination With "Sex or Gender" Attribution (PRESTO 2013-2023)

	Overall[a,b,c] (n=6,578)			Non-Hispanic White Participants[a,b,c] (n=5,701)			BIPOC Participants[a,b,c,d] (n=877)		
	**Preg-nancies**	**Cycles**	**Fully Adjusted FR (95% CI)[e]**	**Preg-nancies**	**Cycles**	**Fully Adjusted FR (95% CI)[f]**	**Preg-nancies**	**Cycles**	**Fully Adjusted FR (95% CI)[f]**
**EVERYDAY DISCRIMINATION**									
**Summary Variable x Attribution[g]**									
None (Score 0)	130	721	1.00 (Reference)	120	626	1.00 (Reference)	10	95	1.00 (Reference)
Low (Score 1-2) + Any attribution other than "sex or gender"	189	1,181	0.91 (0.75-1.12)	171	1,034	0.90 (0.73-1.10)	18	147	1.38 (0.65-2.91)
Medium (Score 3-4) + Any attribution other than "sex or gender"	178	1,194	0.89 (0.73-1.10)	146	952	0.87 (0.70-1.08)	32	242	1.40 (0.70-2.79)
High (Score 5-6) + Any attribution other than "sex or gender"	124	873	0.88 (0.70-1.10)	98	715	0.79 (0.62-1.01)	26	158	1.84 (0.91-3.74)
Very High (Score ≥7) + Any attribution other than "sex or gender"	120	962	0.75 (0.60-0.95)	87	746	0.67 (0.52-0.86)	33	216	1.60 (0.80-3.20)
Low (Score 1-2) + "Sex or gender" attribution	754	4,168	0.99 (0.84-1.18)	682	3,633	0.97 (0.82-1.16)	72	535	1.51 (0.78-2.91)
Medium (Score 3-4) + "Sex or gender" attribution	939	5,516	0.96 (0.81-1.13)	850	4,851	0.94 (0.79-1.11)	89	665	1.36 (0.71-2.58)
High (Score 5-6) + "Sex or gender" attribution	702	4,522	0.88 (0.75-1.05)	601	3,888	0.84 (0.70-1.00)	101	634	1.61 (0.85-3.07)
Very High (Score ≥7) + "Sex or gender" attribution	584	4,255	0.81 (0.68-0.96)	478	3,487	0.76 (0.63-0.91)	106	768	1.47 (0.77-2.80)
**LIFETIME DISCRIMINATION**									
**Number of Experiences x Attribution**									
0	1,721	10,128	1.00 (Reference)	1,541	9,058	1.00 (Reference)	180	1,070	1.00 (Reference)
1 + Any attribution other than "sex or gender"	230	1,557	0.94 (0.83-1.07)	193	1,332	0.92 (0.81-1.06)	37	225	1.02 (0.73-1.41)
2 + Any attribution other than "sex or gender"	96	777	0.81 (0.67-0.98)	81	583	0.88 (0.71-1.08)	15	194	0.50 (0.30-0.82)
≥3 + Any attribution other than "sex or gender"	65	487	0.90 (0.71-1.13)	49	355	0.93 (0.71-1.21)	16	132	0.74 (0.46-1.21)
1 + "Sex or gender" attribution	812	5,120	0.95 (0.88-1.03)	723	4,404	0.98 (0.90-1.06)	89	716	0.75 (0.59-0.95)
2 + "Sex or gender" attribution	477	3,070	0.95 (0.87-1.05)	403	2,486	0.98 (0.89-1.09)	74	584	0.74 (0.57-0.95)
≥3 + "Sex or gender" attribution	345	2,417	0.92 (0.83-1.03)	270	1,868	0.93 (0.82-1.05)	75	549	0.83 (0.64-1.07)

Note: CI = confidence interval; FR = fecundability ratio; PRESTO = Pregnancy Study Online; [a]Limited to participants that reported ever experiencing everyday or lifetime discrimination; [b]Excludes participants that reported ever experiencing everyday discrimination and did not report an attribution when modeled in regression analyses; [c]Excludes participants that reported ever experiencing lifetime discrimination and did not report an attribution when modeled in regression analyses; [d]Includes non-Hispanic Black, Hispanic, non-Hispanic Other (defined as participant self-identified as Asian or Pacific Islander, American Indian or Alaskan Native, multiple races, or some other race); **[e]Adjusted for age, race/ethnicity, parental education, adverse childhood experiences, and childhood financial hardship; [f]Adjusted for age, parental education, adverse childhood experiences, and childhood financial hardship;** [g]Summary variable for everyday discrimination (disrespect, poor service, not smart, afraid of you, harassed) created after assigning a score to each Likert scale and summing across (range: 0-25, where 0 = "never," 1 = "less than once a year," 2 = "a few times a year," 3 = "a few times a month," 4 = "at least once a week," and 5 = "almost every day")

© 2025 Ukah UV et al. *JAMA Network Open*.

eTable 8. Fecundability in Relation to Everyday and Lifetime Discrimination With Response Types (PRESTO 2013-2023)

	Overall[a,b,c]			Excludes Non-Hispanic White participants that reported "race or ethnicity" as an attribution		
	(n=5,597)			(n=5,305)		
	**Pregnancies**	**Cycles**	**Fully Adjusted FR (95% CI)[d]**	**Pregnancies**	**Cycles**	**Fully Adjusted FR (95% CI)[d]**
**EVERYDAY DISCRIMINATION**						
**Summary Variable x Response[e]**						
None (Score 0)	130	721	1.00 (Reference)	108	588	1.00 (Reference)
Low (Score 1-2) + Quiet/Accept	356	2,033	0.98 (0.82-1.18)	326	1,840	0.98 (0.80-1.19)
Medium (Score 3-4) + Quiet/Accept	411	2,527	0.94 (0.79-1.12)	398	2,461	0.92 (0.76-1.11)
High (Score 5-6) + Quiet/Accept	284	2,045	0.82 (0.68-0.99)	273	1,943	0.81 (0.66-1.00)
Very High (Score ≥7) + Quiet/Accept	269	2,111	0.76 (0.63-0.92)	251	1,989	0.74 (0.60-0.91)
Low (Score 1-2) + Talk/Act	232	1,300	0.97 (0.80-1.17)	218	1,206	0.96 (0.78-1.18)
Medium (Score 3-4) + Talk/Act	272	1,599	0.95 (0.79-1.15)	265	1,543	0.94 (0.77-1.16)
High (Score 5-6) + Talk/Act	224	1,304	0.97 (0.80-1.18)	217	1,278	0.94 (0.76-1.16)
Very High (Score ≥7) + Talk/Act	179	1,267	0.85 (0.69-1.05)	172	1,206	0.84 (0.67-1.05)
**LIFETIME DISCRIMINATION**						
**Number of Experiences x Response**						
0	1,721	10,128	1.00 (Reference)	1,628	9,629	1.00 (Reference)
1 + Quiet/Accept	349	2,319	0.92 (0.83-1.03)	321	2,197	0.91 (0.81-1.01)
2 + Quiet/Accept	189	1,304	0.92 (0.80-1.06)	182	1,230	0.95 (0.82-1.09)
≥3 + Quiet/Accept	153	1,177	0.88 (0.75-1.03)	144	1,086	0.89 (0.76-1.05)
1 + Talk/Act	292	1,924	0.93 (0.83-1.04)	275	1,785	0.94 (0.84-1.06)
2 + Talk/Act	175	1,030	1.04 (0.90-1.19)	170	1,006	1.04 (0.90-1.20)
≥3 + Talk/Act	107	702	0.98 (0.81-1.17)	101	651	0.99 (0.82-1.20)

Note: CI = confidence interval; FR = fecundability ratio; PRESTO = Pregnancy Study Online; [a]Limited to participants that reported ever experiencing everyday or lifetime discrimination; [b]Excludes participants that reported ever experiencing everyday discrimination and had a moderately involved response to discrimination (defined as "talk and accept" or "quiet and act") (n=2,090) when modeled in regression analyses; [c]Excludes participants that reported ever experiencing lifetime discrimination and had a moderately involved response to discrimination (defined as "talk and accept" or "quiet and act") (n=1,196) when modeled in regression analyses;
[d]Adjusted for age, race/ethnicity, parental education, adverse childhood experiences, and childhood financial hardship;
[e]Summary variable for everyday discrimination (disrespect, poor service, not smart, afraid of you, harassed) created after assigning a score to each Likert scale and summing across (range: 0-25, where 0 = "never," 1 = "less than once a year," 2 = "a few times a year," 3 = "a few times a month," 4 = "at least once a week," and 5 = "almost every day")

© 2025 Ukah UV et al. *JAMA Network Open*.

eTable 9. Fecundability in Relation to Everyday and Lifetime Discrimination Stratified by Timing of Completion of LCEQ (PRESTO 2013-2023)

	Participants with Retrospective Completion of the LCEQ (n=3,299)			Participants with Prospective Completion of the LCEQ (n=3,279)		
	**Pregnancies**	**Cycles**	**Fully Adjusted FR (95% CI)[a]**	**Pregnancies**	**Cycles**	**Fully Adjusted FR (95% CI)[a]**
**EVERYDAY DISCRIMINATION**						
**Type of Experience[b]**						
Disrespect						
Never	861	4,991	1.00 (Reference)	820	4,624	1.00 (Reference)
Less than once a year	675	4,063	0.97 (0.88-1.06)	655	4,100	0.97 (0.88-1.06)
A few times a year	594	3,932	0.90 (0.81-0.99)	548	3,391	0.96 (0.87-1.07)
≥A few times a month	185	1,545	0.74 (0.64-0.86)	163	1,170	0.86 (0.74-1.01)
Poor service						
Never	1,523	8,996	1.00 (Reference)	1,505	8,870	1.00 (Reference)
Less than once a year	540	3,549	0.93 (0.85-1.02)	462	3,024	0.97 (0.88-1.07)
A few times a year	222	1,708	0.81 (0.71-0.93)	197	1,232	1.00 (0.87-1.15)
≥A few times a month	30	278	0.72 (0.51-1.00)	22	159	0.98 (0.66-1.45)
Not smart						
Never	863	5,121	1.00 (Reference)	783	4,537	1.00 (Reference)
Less than once a year	594	3,595	0.99 (0.90-1.09)	560	3,328	1.00 (0.91-1.10)
A few times a year	650	4,117	0.95 (0.87-1.05)	673	4,310	0.94 (0.86-1.04)
≥A few times a month	208	1,698	0.77 (0.66-0.89)	170	1,110	0.95 (0.81-1.11)
Afraid of you						
Never	1,979	12,046	1.00 (Reference)	1,894	11,176	1.00 (Reference)
Less than once a year	195	1,365	0.93 (0.82-1.07)	173	1,214	0.88 (0.77-1.02)
A few times a year	103	864	0.77 (0.64-0.93)	91	641	0.89 (0.73-1.08)
≥A few times a month	38	256	0.99 (0.73-1.34)	28	254	0.69 (0.48-0.98)
Harassed						
Never	1,112	6,679	1.00 (Reference)	1,020	5,906	1.00 (Reference)
Less than once a year	713	4,513	0.96 (0.88-1.05)	730	4,431	0.98 (0.90-1.07)
A few times a year	384	2,429	0.98 (0.88-1.09)	377	2,423	0.94 (0.84-1.04)
≥A few times a month	106	910	0.76 (0.63-0.92)	59	525	0.70 (0.54-0.90)
**Summary Variable[c]**						
None (Score 0)	449	2,597	1.00 (Reference)	396	2,136	1.00 (Reference)
Low (Score 1-2)	478	2,681	1.00 (0.89-1.13)	491	2,794	1.00 (0.89-1.12)
Medium (Score 3-4)	570	3,396	0.98 (0.88-1.10)	563	3,411	0.96 (0.85-1.08)
High (Score 5-6)	431	2,766	0.92 (0.81-1.04)	405	2,690	0.90 (0.79-1.02)
Very High (Score ≥7)	387	3,091	0.77 (0.67-0.87)	331	2,254	0.88 (0.77-1.01)
**LIFETIME DISCRIMINATION**						
**Any Experience**						
No	1,250	7,236	1.00 (Reference)	1,186	6,904	1.00 (Reference)
Yes	1,065	7,295	0.89 (0.82-0.97)	1,000	6,381	0.97 (0.90-1.05)
**Type of Experience[b,d]**						
On the job	763	5,370	0.89 (0.82-0.97)	706	4,504	0.99 (0.91-1.07)
In housing	146	1,147	0.87 (0.75-1.02)	123	764	1.07 (0.90-1.26)
By police	134	883	1.01 (0.86-1.20)	130	887	0.99 (0.84-1.17)
In the courts	60	469	0.90 (0.70-1.16)	51	311	1.13 (0.87-1.46)
At school	505	3,508	0.92 (0.83-1.01)	457	2,850	1.04 (0.94-1.14)
Getting medical care	287	2,198	0.83 (0.74-0.93)	316	2,140	0.93 (0.83-1.04)
**Number of Experiences**						
0	1,250	7,236	1.00 (Reference)	1,186	6,904	1.00 (Reference)
1	540	3,467	0.93 (0.84-1.02)	521	3,315	0.96 (0.87-1.05)
2	311	2,185	0.88 (0.78-0.99)	269	1,716	0.97 (0.86-1.10)
≥3	214	1,643	0.82 (0.71-0.94)	210	1,350	1.03 (0.90-1.19)

© 2025 Ukah UV et al. *JAMA Network Open*.

Note: CI = confidence interval; FR = fecundability ratio; PRESTO = Pregnancy Study Online; LCEQ = supplemental Life Course Experiences Questionnaire; [a]**Adjusted for age, race/ethnicity, parental education, adverse childhood experiences, and childhood financial hardship;** [b]Not mutually exclusive; [c]Summary variable for everyday discrimination (disrespect, poor service, not smart, afraid of you, harassed) created after assigning a score to each Likert scale and summing across (range: 0-25, where 0 = "never," 1 = "less than once a year," 2 = "a few times a year," 3 = "a few times a month," 4 = "at least once a week," and 5 = "almost every day"); [d]Exposure referent = No to that type of discrimination

© 2025 Ukah UV et al. *JAMA Network Open*.

eTable 10. Fecundability in Relation to Everyday and Lifetime Discrimination Stratified by Pregnancy Attempt Time at Enrollment (PRESTO 2013-2023)

	<3 Cycles of Attempt Time at Enrollment			3-6 Cycles of Attempt Time at Enrollment		
	(n=4,766)			(n=1,812)		
	**Pregnancies**	**Cycles**	**Fully Adjusted FR (95% CI)[a]**	**Pregnancies**	**Cycles**	**Fully Adjusted FR (95% CI)[a]**
**EVERYDAY DISCRIMINATION**						
**Type of Experience[b]**						
Disrespect						
Never	1,319	7,052	1.00 (Reference)	362	2,563	1.00 (Reference)
Less than once a year	1,055	5,931	0.99 (0.92-1.06)	275	2,232	0.88 (0.76-1.02)
A few times a year	875	5,303	0.92 (0.85-1.00)	267	2,020	0.94 (0.81-1.09)
≥A few times a month	270	1,893	0.82 (0.73-0.93)	78	822	0.71 (0.56-0.90)
Poor service						
Never	2,384	13,021	1.00 (Reference)	644	4,845	1.00 (Reference)
Less than once a year	771	4,696	0.94 (0.87-1.02)	231	1,877	0.96 (0.83-1.10)
A few times a year	327	2,163	0.88 (0.79-0.98)	92	777	0.94 (0.76-1.17)
≥A few times a month	37	299	0.77 (0.57-1.04)	15	138	0.90 (0.55-1.47)
Not smart						
Never	1,288	7,053	1.00 (Reference)	358	2,605	1.00 (Reference)
Less than once a year	913	5,078	1.00 (0.93-1.08)	241	1,845	0.99 (0.85-1.16)
A few times a year	1,020	6,073	0.94 (0.87-1.02)	303	2,354	0.97 (0.84-1.12)
≥A few times a month	298	1,975	0.89 (0.79-1.00)	80	833	0.72 (0.57-0.91)
Afraid of you						
Never	3,028	16,884	1.00 (Reference)	845	6,338	1.00 (Reference)
Less than once a year	284	1,808	0.92 (0.83-1.03)	84	771	0.87 (0.70-1.07)
A few times a year	155	1,126	0.84 (0.72-0.98)	39	379	0.77 (0.56-1.05)
≥A few times a month	52	361	0.84 (0.65-1.08)	14	149	0.82 (0.50-1.37)
Harassed						
Never	1,668	9,200	1.00 (Reference)	464	3,385	1.00 (Reference)
Less than once a year	1,125	6,507	0.98 (0.91-1.05)	318	2,437	0.96 (0.84-1.10)
A few times a year	597	3,445	0.98 (0.90-1.07)	164	1,407	0.89 (0.75-1.06)
≥A few times a month	129	1,027	0.76 (0.64-0.90)	36	408	0.67 (0.47-0.93)
**Summary Variable[c]**						
None (Score 0)	671	3,527	1.00 (Reference)	174	1,206	1.00 (Reference)
Low (Score 1-2)	771	4,035	1.01 (0.92-1.11)	198	1,440	0.98 (0.81-1.18)
Medium (Score 3-4)	858	4,841	0.97 (0.88-1.06)	275	1,966	1.00 (0.84-1.19)
High (Score 5-6)	651	3,976	0.91 (0.82-1.00)	185	1,480	0.91 (0.75-1.11)
Very High (Score ≥7)	568	3,800	0.85 (0.76-0.94)	150	1,545	0.71 (0.58-0.88)
**LIFETIME DISCRIMINATION**						
**Any Experience**						
No	1,907	10,399	1.00 (Reference)	529	3,741	1.00 (Reference)
Yes	1,612	9,780	0.95 (0.89-1.01)	453	3,896	0.88 (0.78-0.99)
**Type of Experience[b,d]**						
On the job	1,156	7,022	0.97 (0.91-1.03)	313	2,852	0.83 (0.73-0.94)
In housing	211	1,384	0.94 (0.83-1.07)	58	527	0.96 (0.75-1.23)
By police	201	1,260	0.99 (0.86-1.13)	63	510	1.07 (0.84-1.37)
In the courts	83	521	1.02 (0.83-1.25)	28	259	0.94 (0.65-1.35)
At school	744	4,458	0.98 (0.91-1.06)	218	1,900	0.93 (0.80-1.08)
Getting medical care	460	3,127	0.86 (0.78-0.94)	143	1,211	0.94 (0.79-1.11)
**Number of Experiences**						
0	1,907	10,399	1.00 (Reference)	529	3,741	1.00 (Reference)
1	839	4,891	0.96 (0.90-1.04)	222	1,891	0.87 (0.75-1.01)
2	445	2,818	0.92 (0.83-1.01)	135	1,083	0.94 (0.78-1.13)
≥3	328	2,071	0.94 (0.84-1.05)	96	922	0.82 (0.66-1.01)

© 2025 Ukah UV et al. *JAMA Network Open*.

Note: CI = confidence interval; FR = fecundability ratio; PRESTO = Pregnancy Study Online; LCEQ = supplemental Life Course Experiences Questionnaire**; [a]Adjusted for age, race/ethnicity, parental education, adverse childhood experiences, and childhood financial hardship;** [b]Not mutually exclusive; [c]Summary variable for everyday discrimination (disrespect, poor service, not smart, afraid of you, harassed) created after assigning a score to each Likert scale and summing across (range: 0-25, where 0 = "never," 1 = "less than once a year," 2 = "a few times a year," 3 = "a few times a month," 4 = "at least once a week," and 5 = "almost every day"); [d]Exposure referent = No to that type of discrimination

© 2025 Ukah UV et al. *JAMA Network Open*.

eTable 11. Fecundability in Relation to Everyday and Lifetime Discrimination Stratified by Country of Residence at Enrollment (PRESTO 2013-2023)

	United States (n=5,533)			Canada (n=1,045)		
	Pregnancies	Cycles	Fully Adjusted FR (95% CI)[a]	Pregnancies	Cycles	Fully Adjusted FR (95% CI)[a]
**EVERYDAY DISCRIMINATION**						
**Type of Experience[b]**						
Disrespect						
Never	1,426	8,257	1.00 (Reference)	255	1,358	1.00 (Reference)
Less than once a year	1,084	6,759	0.96 (0.89-1.03)	246	1,404	1.00 (0.85-1.17)
A few times a year	948	6,156	0.93 (0.86-1.00)	194	1,167	0.93 (0.78-1.10)
≥A few times a month	290	2,305	0.79 (0.70-0.89)	58	410	0.88 (0.67-1.15)
Poor service						
Never	2,536	15,108	1.00 (Reference)	492	2,758	1.00 (Reference)
Less than once a year	812	5,434	0.94 (0.88-1.02)	190	1,139	0.96 (0.82-1.12)
A few times a year	358	2,556	0.90 (0.81-1.00)	61	384	0.90 (0.70-1.15)
≥A few times a month	42	379	0.78 (0.59-1.04)	10	58	0.98 (0.54-1.75)
Not smart						
Never	1,391	8,303	1.00 (Reference)	255	1,355	1.00 (Reference)
Less than once a year	968	5,849	1.01 (0.94-1.09)	186	1,074	0.96 (0.81-1.14)
A few times a year	1,072	7,033	0.94 (0.87-1.01)	251	1,394	0.96 (0.82-1.12)
≥A few times a month	317	2,292	0.88 (0.79-0.99)	61	516	0.68 (0.52-0.89)
Afraid of you						
Never	3,227	19,670	1.00 (Reference)	646	3,552	1.00 (Reference)
Less than once a year	305	2,118	0.94 (0.85-1.05)	63	461	0.77 (0.60-0.98)
A few times a year	159	1,256	0.83 (0.71-0.96)	35	249	0.78 (0.56-1.08)
≥A few times a month	57	433	0.88 (0.69-1.13)	9	77	0.66 (0.35-1.22)
Harassed						
Never	1,794	10,701	1.00 (Reference)	338	1,884	1.00 (Reference)
Less than once a year	1,197	7,500	0.98 (0.91-1.04)	246	1,444	0.95 (0.81-1.10)
A few times a year	618	4,077	0.94 (0.87-1.03)	143	775	1.04 (0.86-1.24)
≥A few times a month	139	1,199	0.76 (0.65-0.90)	26	236	0.68 (0.46-1.00)
**Summary Variable[c]**						
None (Score 0)	714	4,061	1.00 (Reference)	131	672	1.00 (Reference)
Low (Score 1-2)	825	4,764	1.00 (0.91-1.09)	144	711	1.04 (0.84-1.29)
Medium (Score 3-4)	929	5,590	0.99 (0.90-1.08)	204	1,217	0.91 (0.75-1.11)
High (Score 5-6)	682	4,602	0.89 (0.81-0.98)	154	854	0.96 (0.77-1.19)
Very High (Score ≥7)	598	4,460	0.84 (0.75-0.93)	120	885	0.76 (0.60-0.97)
**LIFETIME DISCRIMINATION**						
**Any Experience**						
No	2,062	12,130	1.00 (Reference)	374	2,010	1.00 (Reference)
Yes	1,686	11,347	0.93 (0.88-0.99)	379	2,329	0.92 (0.80-1.05)
**Type of Experience[b,d]**						
On the job	1,203	8,193	0.94 (0.88-1.00)	266	1,681	0.91 (0.79-1.04)
In housing	203	1,498	0.93 (0.81-1.06)	66	413	1.01 (0.80-1.28)
By police	229	1,522	1.03 (0.91-1.17)	35	248	0.93 (0.68-1.28)
In the courts	99	696	1.01 (0.84-1.23)	12	84	0.89 (0.52-1.53)
At school	776	5,193	0.98 (0.91-1.05)	186	1,165	0.92 (0.78-1.08)
Getting medical care	495	3,586	0.88 (0.81-0.97)	108	752	0.82 (0.67-0.99)
**Number of Experiences**						
0	2,062	12,130	1.00 (Reference)	374	2,010	1.00 (Reference)
1	871	5,670	0.94 (0.88-1.01)	190	1,112	0.94 (0.80-1.11)
2	463	3,204	0.91 (0.83-1.00)	117	697	0.95 (0.79-1.16)
≥3	352	2,473	0.93 (0.83-1.04)	72	520	0.80 (0.62-1.02)

Note: CI = confidence interval; FR = fecundability ratio; PRESTO = Pregnancy Study Online; LCEQ = supplemental Life Course Experiences Questionnaire; [a]**Adjusted for age, race/ethnicity, parental education, adverse childhood experiences, and childhood**

© 2025 Ukah UV et al. *JAMA Network Open*.

financial hardship; [b]Not mutually exclusive; [c]Summary variable for everyday discrimination (disrespect, poor service, not smart, afraid of you, harassed) created after assigning a score to each Likert scale and summing across (range: 0-25, where 0 = “never,” 1 = “less than once a year,” 2 = “a few times a year,” 3 = “a few times a month,” 4 = “at least once a week,” and 5 = “almost every day”); [d]Exposure referent = No to that type of discrimination

© 2025 Ukah UV et al. *JAMA Network Open*.

eTable 12. Fecundability in Relation to Discrimination by June 2020 for LCEQ Completion (PRESTO 2013-2023)

	Participants with LCEQ Completion Before June 1, 2020 (n=3,148)			Participants with LCEQ Completion After June 1, 2020 (n=3,430)		
	Pregnancies	Cycles	Fully Adjusted FR (95% CI)[a]	Pregnancies	Cycles	Fully Adjusted FR (95% CI)[a]
**EVERYDAY DISCRIMINATION**						
**Type of Experience[b]**						
Disrespect						
Never	800	4,709	1.00 (Reference)	881	4,906	1.00 (Reference)
Less than once a year	660	3,889	1.02 (0.93-1.12)	670	4,274	0.92 (0.84-1.01)
A few times a year	567	3,637	0.94 (0.85-1.04)	575	3,686	0.91 (0.83-1.00)
≥A few times a month	187	1,534	0.75 (0.65-0.88)	161	1,181	0.86 (0.73-1.01)
Poor service						
Never	1,446	8,543	1.00 (Reference)	1,582	9,323	1.00 (Reference)
Less than once a year	522	3,292	0.98 (0.90-1.08)	480	3,281	0.91 (0.83-1.01)
A few times a year	212	1,638	0.81 (0.71-0.93)	207	1,302	0.99 (0.86-1.13)
≥A few times a month	34	296	0.75 (0.55-1.04)	18	141	0.91 (0.60-1.39)
Not smart						
Never	814	4,852	1.00 (Reference)	832	4,806	1.00 (Reference)
Less than once a year	570	3,309	1.05 (0.95-1.16)	584	3,614	0.95 (0.87-1.05)
A few times a year	641	4,114	0.96 (0.87-1.05)	682	4,313	0.94 (0.86-1.03)
≥A few times a month	189	1,494	0.81 (0.69-0.94)	189	1,314	0.89 (0.76-1.03)
Afraid of you						
Never	1,886	11,405	1.00 (Reference)	1,987	11,817	1.00 (Reference)
Less than once a year	189	1,277	0.93 (0.81-1.07)	179	1,302	0.88 (0.77-1.02)
A few times a year	105	873	0.76 (0.63-0.92)	89	632	0.91 (0.75-1.12)
≥A few times a month	34	214	0.97 (0.71-1.32)	32	296	0.72 (0.52-1.01)
Harassed						
Never	1,060	6,429	1.00 (Reference)	1,072	6,156	1.00 (Reference)
Less than once a year	688	4,190	1.02 (0.93-1.11)	755	4,754	0.93 (0.85-1.01)
A few times a year	373	2,367	0.98 (0.88-1.10)	388	2,485	0.93 (0.84-1.04)
≥A few times a month	93	783	0.78 (0.64-0.95)	72	652	0.69 (0.55-0.87)
**Summary Variable[c]**						
None (Score 0)	421	2,382	1.00 (Reference)	424	2,351	1.00 (Reference)
Low (Score 1-2)	459	2,678	0.98 (0.87-1.10)	510	2,797	1.02 (0.91-1.15)
Medium (Score 3-4)	536	3,143	1.00 (0.89-1.13)	597	3,664	0.95 (0.85-1.07)
High (Score 5-6)	412	2,659	0.92 (0.81-1.04)	424	2,797	0.88 (0.78-1.00)
Very High (Score ≥7)	386	2,907	0.80 (0.70-0.91)	332	2,438	0.83 (0.73-0.96)
**LIFETIME DISCRIMINATION**						
**Any Experience**						
No	1,211	7,035	1.00 (Reference)	1,225	7,105	1.00 (Reference)
Yes	1,003	6,734	0.92 (0.85-1.00)	1,062	6,942	0.94 (0.87-1.02)
**Type of Experience[b,d]**						
On the job	719	4,907	0.93 (0.86-1.01)	750	4,967	0.94 (0.87-1.03)
In housing	147	1,018	0.96 (0.82-1.12)	122	893	0.94 (0.79-1.11)
By police	119	761	1.05 (0.88-1.24)	145	1,009	0.97 (0.83-1.14)
In the courts	51	456	0.80 (0.62-1.04)	60	324	1.26 (0.98-1.61)
At school	465	3,146	0.93 (0.84-1.03)	497	3,212	1.01 (0.92-1.11)
Getting medical care	256	1,894	0.87 (0.77-0.98)	347	2,444	0.89 (0.80-0.99)
**Number of Experiences**						
0	1,211	7,035	1.00 (Reference)	1,225	7,105	1.00 (Reference)
1	518	3,368	0.94 (0.85-1.03)	543	3,414	0.95 (0.86-1.04)
2	297	2,000	0.92 (0.81-1.03)	283	1,901	0.93 (0.82-1.05)
≥3	188	1,366	0.89 (0.76-1.03)	236	1,627	0.94 (0.82-1.08)

© 2025 Ukah UV et al. *JAMA Network Open*.

Note: CI = confidence interval; FR = fecundability ratio; PRESTO = Pregnancy Study Online; LCEQ = supplemental Life Course Experiences Questionnaire; [a]**Adjusted for age, race/ethnicity, parental education, adverse childhood experiences, and childhood financial hardship;** [b]Not mutually exclusive; [c]Summary variable for everyday discrimination (disrespect, poor service, not smart, afraid of you, harassed) created after assigning a score to each Likert scale and summing across (range: 0-25, where 0 = “never,” 1 = “less than once a year,” 2 = “a few times a year,” 3 = “a few times a month,” 4 = “at least once a week,” and 5 = “almost every day”); [d]Exposure referent = No to that type of discrimination

© 2025 Ukah UV et al. *JAMA Network Open*.

eTable 13. Fecundability in Relation to Discrimination by June 2020 for LCEQ Completion and Race or Ethnicity (PRESTO 2013-2023)

	Non-Hispanic White Participants					
	Participants with LCEQ Completion Before June 1, 2020			Participants with LCEQ Completion After June 1, 2020		
	(n=2,769)			(n=2,932)		
	Pregnancies	Cycles	Fully Adjusted FR (95% CI)[b]	Pregnancies	Cycles	Fully Adjusted FR (95% CI)[b]
**EVERYDAY DISCRIMINATION**						
**Type of Experience[c]**						
Disrespect						
Never	731	4,216	1.00 (Reference)	801	4,336	1.00 (Reference)
Less than once a year	588	3,440	1.00 (0.91-1.11)	584	3,631	0.92 (0.84-1.02)
A few times a year	483	3,013	0.94 (0.84-1.04)	469	2,993	0.89 (0.80-0.99)
≥A few times a month	162	1,359	0.72 (0.62-0.85)	128	926	0.85 (0.71-1.01)
Poor service						
Never	1,331	7,716	1.00 (Reference)	1,432	8,246	1.00 (Reference)
Less than once a year	449	2,836	0.97 (0.88-1.06)	388	2,610	0.89 (0.80-1.00)
A few times a year	162	1,239	0.78 (0.67-0.91)	148	930	0.95 (0.81-1.11)
≥A few times a month	22	237	0.62 (0.41-0.92)	14	100	0.92 (0.57-1.50)
Not smart						
Never	737	4,267	1.00 (Reference)	725	4,103	1.00 (Reference)
Less than once a year	505	2,919	1.03 (0.93-1.14)	516	3,103	0.96 (0.86-1.06)
A few times a year	559	3,561	0.94 (0.85-1.03)	592	3,659	0.93 (0.85-1.03)
≥A few times a month	163	1,281	0.80 (0.68-0.94)	149	1,021	0.87 (0.73-1.03)
Afraid of you						
Never	1,684	10,038	1.00 (Reference)	1,753	10,171	1.00 (Reference)
Less than once a year	161	1,075	0.92 (0.79-1.06)	142	1,027	0.85 (0.73-1.00)
A few times a year	91	734	0.76 (0.62-0.93)	65	474	0.86 (0.68-1.08)
≥A few times a month	28	181	0.96 (0.68-1.36)	22	214	0.64 (0.43-0.95)
Harassed						
Never	952	5,615	1.00 (Reference)	947	5,244	1.00 (Reference)
Less than once a year	604	3,647	1.00 (0.91-1.10)	644	4,013	0.91 (0.83-1.00)
A few times a year	331	2,100	0.96 (0.86-1.08)	335	2,079	0.94 (0.83-1.05)
≥A few times a month	77	666	0.74 (0.60-0.93)	56	550	0.63 (0.48-0.82)
**Summary Variable[d]**						
None (Score 0)	387	2,139	1.00 (Reference)	387	2,113	1.00 (Reference)
Low (Score 1-2)	419	2,367	0.99 (0.87-1.12)	460	2,425	1.04 (0.92-1.17)
Medium (Score 3-4)	481	2,757	1.00 (0.89-1.13)	528	3,128	0.97 (0.86-1.09)
High (Score 5-6)	359	2,338	0.89 (0.78-1.01)	349	2,320	0.86 (0.75-0.99)
Very High (Score ≥7)	318	2,427	0.77 (0.67-0.89)	258	1,900	0.81 (0.70-0.94)
**LIFETIME DISCRIMINATION**						
**Any Experience**						
No	1,103	6,355	1.00 (Reference)	1,092	6,329	1.00 (Reference)
Yes	861	5,673	0.92 (0.85-1.00)	890	5,557	0.97 (0.90-1.06)
**Type of Experience[c,e]**						
On the job	624	4,132	0.95 (0.87-1.04)	625	4,010	0.95 (0.87-1.04)
In housing	122	841	0.94 (0.79-1.11)	92	653	0.95 (0.78-1.15)
By police	88	572	0.99 (0.81-1.20)	105	626	1.07 (0.89-1.29)
In the courts	40	317	0.87 (0.65-1.16)	45	250	1.19 (0.90-1.58)
At school	391	2,576	0.94 (0.85-1.05)	388	2,402	1.02 (0.92-1.13)
Getting medical care	215	1,612	0.84 (0.74-0.96)	293	1,944	0.92 (0.82-1.04)
**Number of Experiences**						
0	1,103	6,355	1.00 (Reference)	1,092	6,329	1.00 (Reference)
1	455	2,923	0.94 (0.85-1.03)	476	2,906	0.97 (0.88-1.07)
2	251	1,625	0.93 (0.82-1.06)	238	1,474	0.98 (0.86-1.11)
≥3	155	1,125	0.87 (0.74-1.03)	176	1,177	0.97 (0.83-1.13)

© 2025 Ukah UV et al. *JAMA Network Open*.

	BIPOC[a] Participants					
	Participants with LCEQ Completion Before June 1, 2020			Participants with LCEQ Completion After June 1, 2020		
	(n=379)			(n=498)		
	Pregnancies	Cycles	Fully Adjusted FR (95% CI)[b]	Pregnancies	Cycles	Fully Adjusted FR (95% CI)[b]
**EVERYDAY DISCRIMINATION**						
**Type of Experience[c]**						
Disrespect						
Never	69	493	1.00 (Reference)	80	570	1.00 (Reference)
Less than once a year	72	449	1.15 (0.84-1.57)	86	643	0.94 (0.71-1.26)
A few times a year	84	624	0.96 (0.71-1.31)	106	693	1.04 (0.79-1.36)
≥A few times a month	25	175	1.00 (0.65-1.53)	33	255	0.89 (0.60-1.31)
Poor service						
Never	115	827	1.00 (Reference)	150	1,077	1.00 (Reference)
Less than once a year	73	456	1.07 (0.81-1.42)	92	671	1.04 (0.81-1.32)
A few times a year	50	399	0.96 (0.70-1.31)	59	372	1.13 (0.86-1.50)
≥A few times a month	12	59	1.32 (0.76-2.28)	4	41	0.89 (0.37-2.16)
Not smart						
Never	77	585	1.00 (Reference)	107	703	1.00 (Reference)
Less than once a year	65	390	1.23 (0.91-1.67)	68	511	0.90 (0.68-1.20)
A few times a year	82	553	1.18 (0.88-1.59)	90	654	0.94 (0.72-1.22)
≥A few times a month	26	213	0.88 (0.57-1.37)	40	293	0.94 (0.66-1.34)
Afraid of you						
Never	202	1,367	1.00 (Reference)	234	1,646	1.00 (Reference)
Less than once a year	28	202	1.00 (0.69-1.45)	37	275	1.01 (0.72-1.41)
A few times a year	14	139	0.87 (0.51-1.48)	24	158	1.11 (0.74-1.66)
≥A few times a month	6	33	0.98 (0.47-2.07)	10	82	1.06 (0.58-1.94)
Harassed						
Never	108	814	1.00 (Reference)	125	912	1.00 (Reference)
Less than once a year	84	543	1.22 (0.93-1.60)	111	741	1.05 (0.83-1.34)
A few times a year	42	267	1.18 (0.84-1.65)	53	406	0.96 (0.71-1.31)
≥A few times a month	16	117	1.06 (0.64-1.75)	16	102	1.02 (0.61-1.72)
**Summary Variable[d]**						
None (Score 0)	34	243	1.00 (Reference)	37	238	1.00 (Reference)
Low (Score 1-2)	40	311	1.12 (0.71-1.74)	50	372	0.95 (0.63-1.42)
Medium (Score 3-4)	55	386	1.12 (0.75-1.69)	69	536	0.85 (0.59-1.25)
High (Score 5-6)	53	321	1.28 (0.85-1.92)	75	477	1.00 (0.69-1.45)
Very High (Score ≥7)	68	480	1.13 (0.75-1.71)	74	538	0.94 (0.64-1.37)
**LIFETIME DISCRIMINATION**						
**Any Experience**						
No	108	680	1.00 (Reference)	133	776	1.00 (Reference)
Yes	142	1,061	0.85 (0.67-1.08)	172	1,385	0.74 (0.60-0.92)
**Type of Experience[c,e]**						
On the job	95	775	0.80 (0.63-1.02)	125	957	0.89 (0.72-1.11)
In housing	25	177	0.88 (0.59-1.34)	30	240	0.86 (0.60-1.23)
By police	31	189	1.43 (0.99-2.06)	40	383	0.72 (0.53-1.00)
In the courts	11	139	0.58 (0.32-1.04)	15	74	1.55 (0.92-2.60)
At school	74	570	0.84 (0.64-1.09)	109	810	0.96 (0.76-1.21)
Getting medical care	41	282	1.02 (0.74-1.40)	54	500	0.72 (0.54-0.96)
**Number of Experiences**						
0	108	680	1.00 (Reference)	133	776	1.00 (Reference)
1	63	445	0.92 (0.69-1.23)	67	508	0.78 (0.59-1.03)
2	46	375	0.75 (0.54-1.04)	45	427	0.65 (0.47-0.90)
≥3	33	241	0.89 (0.61-1.31)	60	450	0.78 (0.58-1.04)

Note: CI = confidence interval; FR = fecundability ratio; PRESTO = Pregnancy Study Online; LCEQ = supplemental Life Course Experiences Questionnaire; [a]Includes non-Hispanic Black, Hispanic, non-Hispanic Other (defined as participant self-identified as

© 2025 Ukah UV et al. *JAMA Network Open*.

Asian or Pacific Islander, American Indian or Alaskan Native, multiple races, or some other race); [b]**Adjusted for age, parental education, adverse childhood experiences, and childhood financial hardship;** [c]Not mutually exclusive; [d]Summary variable for everyday discrimination (disrespect, poor service, not smart, afraid of you, harassed) created after assigning a score to each Likert scale and summing across (range: 0-25, where 0 = "never," 1 = "less than once a year," 2 = "a few times a year," 3 = "a few times a month," 4 = "at least once a week," and 5 = "almost every day"); [e]Exposure referent = No to that type of discrimination

© 2025 Ukah UV et al. *JAMA Network Open*.
