# Supplementary material for: Fecundability in Association With Everyday and Lifetime Discrimination
Source: JAMA Netw Open. 2025 Jul 14;8(7):e2520597. doi: 10.1001/jamanetworkopen.2025.20597 (PMC12260993; doi:10.1001/jamanetworkopen.2025.20597)
Supplement: Supplement 2. — Data Sharing Statement [file jamanetwopen-e2520597-s002.pdf]

Data Sharing Statement

Ukah. Fecundability in Association With Everyday and Lifetime Discrimination. *JAMA Netw Open*. Published July 14, 2025. doi:10.1001/jamanetworkopen.2025.20597

Data
Data available: No
